# Molecular Pathology of Pulmonary Large Cell Neuroendocrine Carcinoma: Novel Concepts and Treatments

**DOI:** 10.3389/fonc.2021.671799

**Published:** 2021-04-22

**Authors:** Masayo Yoshimura, Kurumi Seki, Andrey Bychkov, Junya Fukuoka

**Affiliations:** ^1^ Department of Pathology, Kameda Medical Center, Kamogawa, Japan; ^2^ Department of Pathology, Nagasaki University Graduate School of Biomedical Sciences, Nagasaki, Japan

**Keywords:** large cell neuroendocrine carcinoma, lung, DLL3, ASCL1, RB1, TP53

## Abstract

Pulmonary large cell neuroendocrine carcinoma (LCNEC) is an aggressive neoplasm with poor prognosis. Histologic diagnosis of LCNEC is not always straightforward. In particular, it is challenging to distinguish small cell lung carcinoma (SCLC) or poorly differentiated carcinoma from LCNEC. However, histological classification for LCNEC as well as their therapeutic management has not changed much for decades. Recently, genomic and transcriptomic analyses have revealed different molecular subtypes raising hopes for more personalized treatment. Two main molecular subtypes of LCNEC have been identified by studies using next generation sequencing, namely type I with *TP53* and *STK11*/*KEAP1* alterations, alternatively called as non-SCLC type, and type II with *TP53* and *RB1* alterations, alternatively called as SCLC type. However, there is still no easy way to classify LCNEC subtypes at the actual clinical level. In this review, we have discussed histological diagnosis along with the genomic studies and molecular-based treatment for LCNEC.

## Introduction

Neuroendocrine tumors (NET) of the lung account for approximately 20% of all lung cancers with a majority being represented by small cell lung cancer (SCLC) (1–3). Large cell neuroendocrine carcinoma (LCNEC) is rare, accounting for 1–3% of all lung cancer cases (4, 5). The increasing incidence has been reported recently, with a rate raised from 0.26 in 2004 to 0.39 per 100,000 people in 2015 (5–7). Most LCNEC patients are male and their median age is 66 (5–7). There is a high frequency of cigarette smoking history, up to 98% (4, 6). LCNEC usually occurs in the lung periphery. Median survival time from diagnosis is 9.7 months and 54.6% of patients have stage IV LCNEC at the time of diagnosis (7). LCNEC is a unique tumor that shows immunohistochemical and morphological traits of both SCLC and non-small cell lung carcinoma (NSCLC). Although LCNEC is currently categorized into NSCLC, due to its overlapping features of SCLC, treatment protocols for NSCLC and SCLC are used depending on the situations. Also with the rarity of the disease, no standard treatment for LCNEC has been currently developed. Recently, two main molecular subtypes of LCNEC have been identified by next generation sequencing (NGS) studies, namely type I with *TP53* and *STK11*/*KEAP1* alterations (NSCLC type), and type II with *TP53* and *RB1* alterations (SCLC type) (8). These subtypes are currently considered as relevant for prognosis and selecting the therapeutic regimen. In this review, we will first introduce current histopathological features of LCNEC and its criteria to distinguish from other histological types. And then, we will focus on the molecular subtypes and their influence on pathological diagnosis. The emphasis will be done on immunohistochemical subgrouping added by the concise review of studies about treatment for LCNEC based on molecular subtype.

## Current Pathological Diagnosis of LCNEC

**Figure 1:**
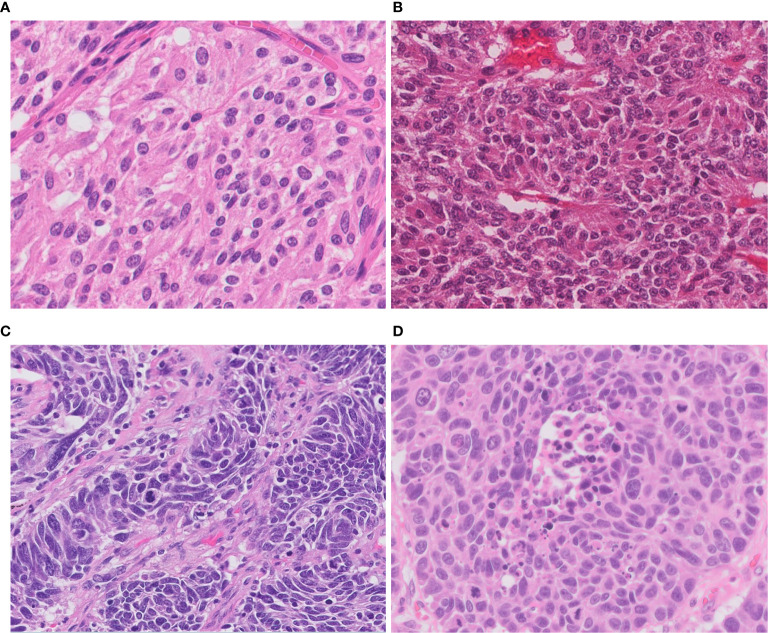
Morphology of neuroendocrine tumors of the lung. **(A)** Typical carcinoid shows solid nests with zellballen patterns; the tumor cells are uniform with a moderate amount of eosinophilic cytoplasm. **(B)** Atypical carcinoid with rosette formation. **(C)** SCLC showing sheets of small cells with scant cytoplasm, finely granular chromatin, and mitoses. **(D)** LCNEC displays organoid nesting and palisading patterns; tumor cells have abundant eosinophilic cytoplasm, coarsely granular chromatin, and prominent nucleoli. Magnification: ×40.

The World Health Organization (WHO) 2015 classification of malignant pulmonary neuroendocrine tumors includes four major histological types: low-grade typical carcinoid, intermediate-grade atypical carcinoid, high-grade SCLC and LCNEC (1). There are tumorlets and diffuse idiopathic pulmonary neuroendocrine cell hyperplasia (DIPNECH) in the category of neuroendocrine cell tumors, but both are currently considered to be the pre-invasive benign lesions. All of these neoplasms show various levels of organoid growth patterns (rosette, palisading, trabeculae, ribbons, festoons, lobular nests), they are distinguished conventionally based on hematoxylin and eosin (H&E) features such as mitotic rate, presence of necrosis, and cytologic details including the presence of large nucleoli and abundant cytoplasm (**Table 1**, **Figure 1**). LCNEC is defined as a NSCLC with neuroendocrine morphology and the expression of neuroendocrine markers (1). LCNEC cells are typically more than three times diameter of small lymphocytes and exhibit abundant cytoplasm, nucleoli or often with vesicular chromatin. The use of immunohistochemistry (IHC) for the purpose of detecting neuroendocrine feature, synaptophysin, chromogranin, and CD56, is mandatory.

**Table 1:** Main clinicopathological characteristics of neuroendocrine tumors of the lung.

	DIPNECH	typical carcinoid	atypical carcinoid	SCLC	LCNEC
Clinical characteristics					
Age	50-60	<60 (mean 45yrs)	<60 (mean 55yrs)	mean 67 yrs	mean 66 yrs
Gender predilection	female	no	no	male	male
Risk factor	unrecognized pulmonary injury	MEN1 (<5%)	MEN1 (<5%), smoking	smoking	smoking
Site	periphery	central airways	central airways, more peripheral compared to TC	centrally in the major airways	upper lobe, periphery
Prognosis (5-year survival)	rarely die	rarely die (even with regional metastasis)	71-76%	5%	15-57%
Symptoms	1/3 asymptomatic; 1/2 asthma-like symptoms (cough, wheezing, dyspnea); decreased respiratory function	most are asymptomatic; occasionally carcinoid syndrome, Cushing syndrome, and acromegaly	most are asymptomatic; occasionally carcinoid syndrome, Cushing syndrome, and acromegaly	fatigue, cough, dyspnea, decreased appetite, weight loss, pain and hemoptysis	chest pain with hemoptysis, dyspnea, cough, fever, weight loss; up to 1/4 asymptomatic
**Pathological findings**					
Microscopic findings	NE cell hyperplasia and/or multiple tumorlets	uniform cytologic features; cytoplasm: moderate amount; of eosinophilic; nucleoli: inconspicuous or absent	uniform cytologic features cytoplasm: moderate amount of eosinophilic nucleoli: sometimes seen	small size (less than the diameter of three small lymphocytes); cytoplasm: scant; nuclei: finely granular nuclear chromatin; nucleoli: absent or faint	large size (more than the diameter of three small lymphocytes); cytoplasm: abundant; nuclei: vesicular of fine chromatin; nucleoli: frequent
Necrosis	–	–	+ (often punctate)	+ (frequent)	+
Mitotic rate	<2/10 HPF or 2mm²	<2/10 HPF or 2mm²	2-10/10 HPF or 2mm²	≥11/10 HPF or 2mm² (median:80)	≥11/10 HPF or 2mm² (median:70)
Ki-67 index	low (< 10-20%)	low (< 10-20%)	Low (< 20%)	high (80-100%)	high (> 40%)
**Immunoexpression of neuroendocrine markers**					
Synaptophysin	100%	100%	79-100%	83-100%	80-87%
Chromogranin A	100%	94-100%	79-89%	4.2-47%	9-57%
CD56	74%	60-100%	57-100%	79-100%	36-90%
TTF-1	100%	28-94%	29-100%	77-90%	38-75%
ASCL1	100%	65%	64%	79%	73%

DIPNECH, diffuse idiopathic pulmonary neuroendocrine cell hyperplasia; SCLC, small cell lung cancer; LCNEC, large cell neuroendocrine carcinoma; HPF, high power field.

The primary differential diagnosis for LCNEC include SCLC and other types of NSCLC. LCNEC and SCLC are differentially diagnosed based on cytological appearance including prominent nucleoli, vesicular to clumped versus finely granular chromatin, cell size, and more abundant cytoplasm in LCNEC. NSCLC can be differentiated from LCNEC by absence of neuroendocrine morphology and IHC expression. Basaloid squamous cell carcinoma shows nested growth pattern with peripheral palisading and large areas of necrosis, mimicking LCNEC. Although p40 is useful squamous epithelial marker to distinguish LCNEC from basaloid squamous cell carcinoma, it should be noted that some LCNEC may also show focal staining (<10% tumor cells labeling) (9). The diffuse and strong p40 positivity is characteristic for basaloid squamous cell carcinoma.

**Figure 2:**
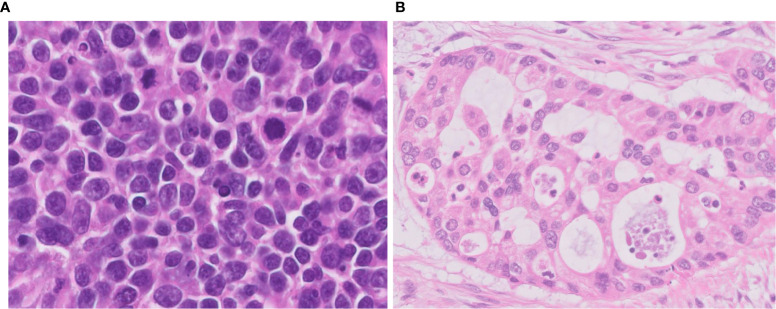
Difficulties in differential diagnosis of LCNEC. **(A)** This tumor is LCNEC that needs to be differentiated from SCLC; cell size is larger than that of SCLC. **(B)** This LCNEC needs to be differentiated from adenocarcinoma; the tumor shows pseudoglandular structures forming cribriform pattern. Magnification: ×40.

The difficulty of distinguishing between LCNEC and SCLC is that there are cases showing borderline features (**Figure 2A**). Use of IHC to distinguish LCNEC and SCLC is with limited benefits. There are currently no entirely sensitive and specific IHC markers to separate LCNEC and SCLC. Some markers including CK7, 8, 18, and 19 were reported to be significantly weaker in SCLC than in LCNEC. In SCLC, these cytokeratins typically show dot-like cytoplasmic staining (10–12), however, similar staining patterns can also be seen in LCNEC. One of the potential useful markers is napsin A. Based on the literature, focal and weak staining can be seen in up to 15% of LCNECs, whereas SCLC is consistently negative for this marker (13, 14). Therefore, there are significant interobserver variability.

**Figure 3:**
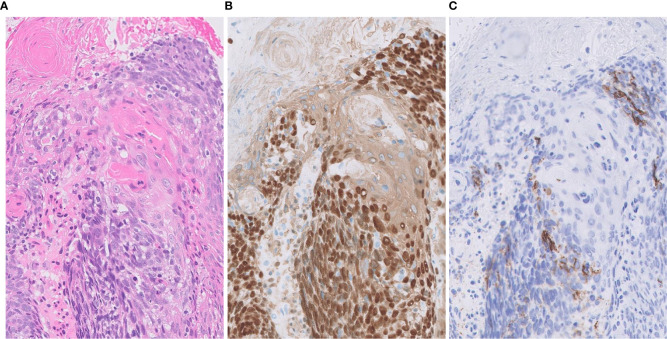
Focal expression of CD56 in squamous cell carcinoma. **(A)** Tumor cells form solid nests with occasional keratinization. **(B)** Diffusely positivity for p40 (nuclear) and CK14 (cytoplasmic). **(C)** Focal expression of neuroendocrine marker CD56. Magnification: ×20.

One of the important pathological judgements outside the pulmonary NETs is distinguishing some types of poorly differentiated NSCLC, which can be difficult to distinguish solely by H&E observation (**Figure 2B**). Usually, it is possible to distinguish between NSCLC and pulmonary NETs by the presence or absence of neuroendocrine marker expression (synaptophysin, chromogranin A, and CD56). However, it is important to know that neuroendocrine markers are not always clear to separate those two types since 15-23% of high-grade neuroendocrine carcinomas are stained negative even with a combination of all three markers and nearly 15% of NSCLCs are positive for one of these neuroendocrine markers (15, 16). Identifying neuroendocrine morphology along with confirming diffuse staining for neuroendocrine markers occupying the majority of the whole tumor area are critical (**Figure 3**).

Recently, a new category of tumors called SMARCA4-deficient undifferentiated thoracic tumor has been reported. These tumors show undifferentiated round cells or rhabdoid morphology (17, 18). Although these tumors do not show neuroendocrine architecture, they are diffusely positive for synaptophysin in 70% of cases (17). They also show geographic necrosis, high mitotic and Ki-67 rate. Therefore, it should be also noted that in a crushed biopsy, these features may closely mimic LCNEC. Other immunostaining is useful to distinguish between the two in such cases. Typical immunohistochemical features for SMARCA4-deficient undifferentiated thoracic tumor is the lack of claudin 4 expression and low or absent keratin immunostaining, in addition to the loss of SMARCA4 (BRG-1) (17–20).

**Figure 4:**
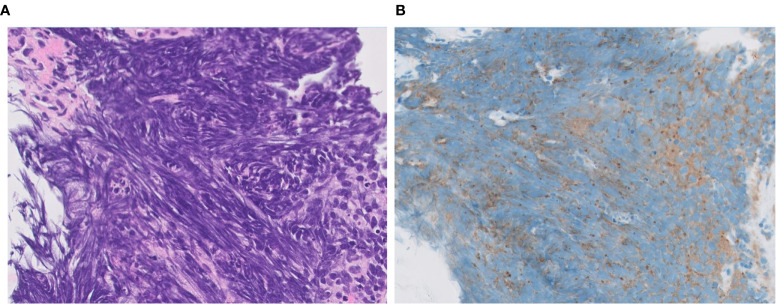
Extensive crush artifact in small cell lung carcinoma. SCLC is often crushed during biopsy, which affects appearance on routine staining (**A**, H&E) and immunohistochemistry (**B**, synaptophysin). Magnification: ×40.

Recently, several new immunohistochemical markers, such as Insulinoma-associated protein 1 (INSM1) and Achaete-scute homolog-1 (ASCL1) have been reported as neuroendocrine markers with nuclear expression (16, 21–27). INSM1 has been reported to have excellent sensitivity and specificity for the diagnosis of lung NETs (26, 27), and no cases of NSCLC have reported to be positive for ASCL1 immunostaining so far (16). An important benefit of these markers is their staining localization. Nuclear staining is suggested optimal for lung NET diagnosis since many specimens of these tumors obtained by transbronchial biopsy show strong crush artifacts gotten at the time of sampling (**Figure 4**) and, for that occasion, evaluation of cytoplasmic (synoptophysin, chromagranin A) and membranous (CD56) immunoexpression is difficult.

**Figure 5:**
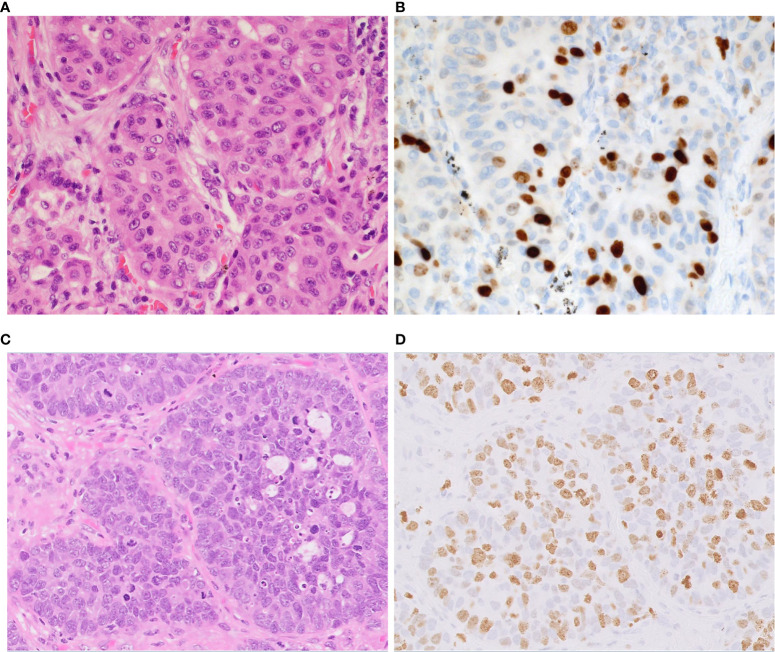
Proliferation index in atypical carcinoid vs. LCNEC. Representative examples of atypical carcinoid **(A, B)** and LCNEC **(C, D)**. **(A)** The tumor shows nests of carcinoid tumor cells. **(B)** The Ki-67 proliferative index is approximately 20%. **(C)** The tumor shows nests and palisading patterns of LCNEC tumor cells. **(D)** The Ki-67 proliferative index is approximately 60%. Magnification: ×40.

Extrapulmonary NETs are graded as G1, G2, or G3 based on mitotic count, Ki-67 labeling index (LI) and presence of necrosis (28). Because Ki-67 LI is overlapped among different grades in pulmonary NETs without clear cutoff, the main utility of this biomarker is to distinguish the carcinoids from high grade pulmonary NETs (29). Ki-67 LI can be used to separate LCNEC from typical and atypical carcinoid tumors where LCNEC typically display LI >40% whereas that of carcinoid tumors is < 20% (9, 29) (**Figure 5**). Ki-67 LI has been proposed as a prognostic factor in surgically resected specimens of typical and atypical carcinoids, with cutoff values ranging from 2.5% to 5.8% (29). Our group showed that scoring hot sports is mandatory for survival prediction in NSCLC (30). However, studies exploring prognostic utility of Ki-67 in LCNEC could not find statistically significant correlation between LI and overall survival and disease-free survival (31, 32). Additional well-powered studies with standardized scoring approach are needed to elucidate a role of Ki-67 as diagnostic and/or prognostic biomarker in LCNEC.

**Figure 6:**
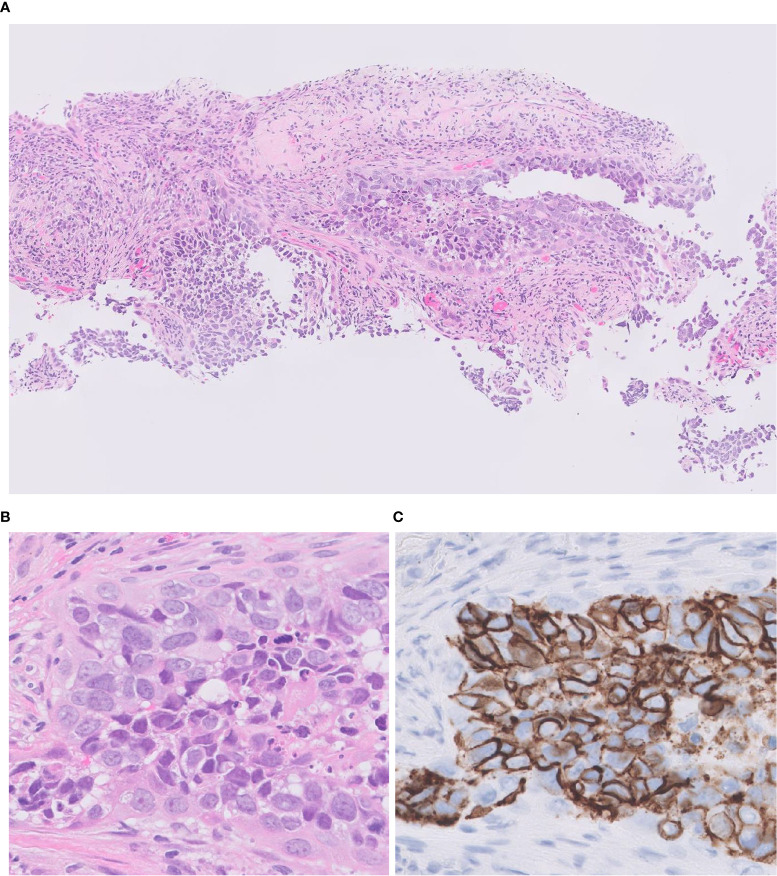
Transbronchial lung biopsy of LCNEC. A small-sized specimen featuring tumor cells with abundant eosinophilic cytoplasm, coarsely granular chromatin, and prominent nucleoli. H&E **(A, B)**; CD 56 **(C)** Magnification: ×10 **(A)**; ×40 **(B, C)**.

The histological diagnosis of LCNEC is challenging in small biopsy specimens unless a full-developed morphology consistent with LCNEC diagnosis is disclosed. The major reason is tissue heterogeneity of the tumor due to admixture of other patterns (32). Therefore, WHO does not recommend rendering pathological diagnosis of LCNEC on the small biopsies, instead the diagnosis should be limited to surgically removed specimens. However, considering the unignorable frequency of advance disease status at the time of presentation and the importance of histological diagnosis to select the best therapeutic regimen, the improvement of accurate diagnosis of LCNEC for the small biopsies such as transbronchial lung biopsy, needle biopsy, and cryobiopsy is important. There indeed are several cases showing convincing morphological and IHC features to make diagnosis of LCNEC (**Figure 6**). Accumulating evidence to prove diagnostic feasibility for biopsy samples with combination of H&E and IHC panels is mandatory. For those occasions, molecular classifiers including new IHC markers described in below sections may be useful.

## Molecular Features

Genetic background of LCNEC has been widely studied, particularly, with a reference to molecular signature of NSCLC. The common molecular alterations are shown in **Table 2**. Among tumor suppressor genes altered in LCNEC, the *TP53* mutation is the most common (64-92%), followed by the *RB1* mutation (19-42%) (**Table 2**) (8, 9, 33–44).

**Table 2:** Molecular alterations in LCNEC.

	Gene	Prevalence	References
Gene mutations			
Cell cycle	*TP53*	64–92%	(8, 9, 33–44)
	*RB1*	19–42%	(8, 9, 34–44)
	*CDKN2A*	4–8%	(8, 9, 45)
MAPK pathway	*STK11*	17–33%	(8, 9, 33, 35–37, 39, 40, 44)
	*KEAP1*	19–31%	(8, 9, 35, 36, 40, 44, 45)
Cell adhesion	*ADAMTS12*	20%	(8, 9, 45)
	*ASAMTS2*	15%	(8)
Neurogenesis	*GAS7*	12%	(8)
	*NTM*	10%	(8)
Notch pathway	*NOTCH1*	10–16%	(8, 34, 38, 39, 44, 45)
	*NOTCH2*	4–7%	(8, 34, 38, 40, 41, 43, 45)
	*NOTCH3*	4–6%	(8, 34, 38, 43, 45)
	*NOTCH4*	6–8%	(8, 38, 45)
Driver genes	*KRAS*	4–24%	(9, 34, 39, 46)
	*EGFR*	0–4%	(9, 34, 39, 46)
	*ALK*	0–2%	(34, 46)
	*RET*	2%	(46)
PI3K-AKT-mTOR pathway	*PTEN*	4–5%	(34, 45)
	*PIK3CA*	3%	(34, 45)
	*AKT2*	4%	(34, 45)
	*PICTOR*	5%	(34)
	*mTOR*	1%	(34)
	*NF1*	5%	(45)
	*INSR*	3%	(45)
	*TSC2*	2%	(45)
	*PRCTR*	2%	(45)
**Gene amplifications**			
	*NKX2-1*	10–20%	(8, 9, 45)
	*MYC*	5–13%	(8, 9, 45)
	*MYCL1*	9–12%	(8, 45)
	*SOX2*	3–11%	(9, 45)
	*FGFR*	3–7%	(8, 45)
	*IRS2*	3–4%	(8, 9, 45)
	*MYCN*	1–2%	(9, 45)

The most common alterations in driver genes are *KRAS* mutations (4-24%), with a much less frequency of *EGFR*, *ALK*, *RET*, and absence of others driver mutations associated with NSCLC, such as *BRAF* and *ROS1* (9, 34, 39, 46). Genomic mutations associated with the PI3K-AKT-mTOR pathway are found in LCNECs, including *PTEN* (4–5%), *PIK3CA* (3%), *AKT2* (4%), *PICTOR* (5%), *mTOR*: (1%), *NF1* (5%), *INSR* (3%), *TSC2* (2%), and *PRCTR* (2%) (34, 45). Amplifications in NK2 homeobox 1 (*NKX2-1*, also known as TTF-1; 10–20%), v-myc avian myelocytomatosis viral oncogene homolog gene (*MYC*; 5-13%), v-myc avian myelocytomatosis viral oncogene lung carcinoma derived homolog gene (*MYCL1*; 12%), SRY-box 2 gene (*SOX2*; 11%), fibroblast growth factor receptor 1 (*FGFR1*; 4–7%), insulin receptor substrate 2 (*IRS2*; 3–4%) and v-myc avian myelocytomatosis viral oncogene neuroblastoma derived homolog gene (*MYCN*; 2%) have also been identified. Cyclin-dependent kinase inhibitor 2A (*CDKN2A*) deletions were reported with a frequency of 4% to 8% (8, 9, 45).

In addition to gene mutations, chromosomal alterations have been reported in LCNEC; in particular, alterations greater than 10 Mb, losses of 1p, 3p, 4p, 4q, 5q, 8p, 10q, 13q, 17p and gains of 3q, 5p, 8q, 18q, were found to be much more frequent in LCNECs as compared to pulmonary carcinoid tumors (47).

## Molecular Subtypes

George et al. reported the existence of two LCNEC genomic subtypes with specific transcriptional patterns, which they categorized as type I (NSCLC type) with *TP53* and *STK11/KEAP1* alterations and type II (SCLC type) with *TP53* and *RB1* alterations, which is introduced in the upcoming 2021 WHO classification (8). Although type I LCNEC shares genomic alterations with pulmonary adenocarcinomas and squamous cell carcinomas, no transcriptional relationship was found, and it was divided into transcriptional subgroups (ASCL1^high^/DLL3^high^/NOTCH^low^) with similarity to SCLC. *TP53* and *RB1* are tumor suppressor genes, and mutations have been reported in LCNEC as well as in SCLC. One of the hallmarks of SCLC is bi-allelic alterations in *TP53* and *RB1* (48, 49). While type II LCNECs reveals genetic resemblance to SCLC, these tumors are markedly different from SCLC transcriptional subgroups (ASCL1^low^/DLL3^low^/NOTCH^high^). Therefore, the distinction between type I and type II LCNECs from SCLC is important to be able to evaluate the response of patients to treatment options.

### Major Genes Involved in Transcriptional Signatures of LCNEC

Serine/threonine kinase 11 (*STK11*) encodes liver kinase B1 (LKB1) and is a commonly altered tumor suppressor that frequently occurs in NSCLC (50). LKB1 directly phosphorylates and activates adenosine monophosphate-activated protein kinase (AMPK) (51). In response to energetic stress, AMPK alters the cellular metabolism to restore nicotinamide adenine dinucleotide phosphate (NADPH) concentrations (52). It also regulates the activity of mTOR. Under energetic stress, the LKB1-AMPK axis plays a critical role in modulating cell growth and proliferation to maintain adequate ATP and NADPH levels (53).

Kelch-like ECH-associated protein 1 (KEAP1) forms a protein complex and ubiquitinates the N-terminal domain of NRF2, an oxidative stress-responsive transcription factor (54). Oxidative stress induces the oxidation of KEAP1 at key cysteine residues which causes a conformational change in KEAP1 releasing NRF2, resulting in translocation and nuclear accumulation of NRF2. In the nucleus, NRF2 forms a heterodimer with its partner sMAF (v-Maf avian musculoaponeurotic fibrosarcoma oncogene homolog) and binds to antioxidant responsive element (ARE) sequences to regulate the transcription of target genes (55). A major NRF2 transcriptional target is NADPH (56, 57). *KEAP1* is not only a tumor suppressor gene, but also a metastasis suppressor gene (58). *KEAP1* mutations co-occur with mutations in *STK11*, which have also been associated with poor response to immune checkpoint blockade in lung adenocarcinoma (59–61). Therefore, *STK11*/*KEAP1* mutations can be expected as predictive biomarkers for anti-PD-1/PD-L1 therapy.

*ASCL1* is a transcription factor and is selectively expressed in normal fetal pulmonary neuroendocrine cells. ASCL1 is highly expressed in SCLC and LCNEC, where it acts to maintain neuroendocrine features (62). The Notch pathway likewise plays an important role in the developing respiratory system and regulates neuroendocrine versus epithelial cell fate decisions (63). Notch genes encode single transmembrane receptors that mediate short-range communication between cells (64, 65). When Notch binds to its ligand (delta-like ligands: DLL1, DLL3 and DLL4, jagged ligands: JAG1 and JAG2) expressed on adjacent cells, Notch receptors (Notch1–4) release the Notch intracellular domain (NICD). NICD activates transcription of HES1 (hairy and enhancer of split 1 and HEY1 [hairy and enhancer of split-related protein 1]), which encodes transcriptional repressors of ASCL1 (66). On the other hand, DLL3 is a transcriptional target of ASCL1 (62, 67). And unlike other Notch ligands (DLL1, DLL4, JAG1 and JAG2), DLL3 without the conserved N-terminal module of agonistic Notch ligands can antagonize DLL1-Notch signaling (68). Thus, ASCL1 both activates Notch signaling and is repressed by it. DLL3 predominantly localizes to the Golgi apparatus, where it retains other Notch members and redirects them to endosomes for degradation (69). Some DLL3 is expressed on the cell surface, which is not expressed in normal lung tissue (70). Thus, DLL3 protein has emerged as a very promising drug target.

### Mutations Associated With LCNEC Subtypes

We reviewed the frequency of mutations associated with molecular subtypes of LCNEC. PubMed was searched for papers with keywords: lung, LCNEC and molecular/genetic alterations. Initially, we retrieved 94 papers. After excluding review papers and case reports, the remaining 17 studies containing information on alterations for *TP53*, *RB1*, *STK11*, *KEAP1*, *NOTCH*, *ASCL1* and *DLL3* were analyzed (8, 9, 24, 33–44, 71, 72). A summary of these studies is shown in **Table 3** (raw data provided in **Supplemental Table 1**).

**Table 3:** Frequency of gene alterations associated with LCNEC molecular subtypes.

Gene/alteration	+ve	-ve	total
***TP53***	**427**	**99**	**526**
mutations	329		
CNV	5		
CAN	2		
LOH	2		
n/s	89		
mutations+(CNV/CNA/LOH)	15		
***RB1***	**224**	**313**	**537**
mutations	131		
CNV	3		
CNA	3		
LOH	4		
homozygous deletion	14		
n/s	69		
***STK11***	**70**	**340**	**410**
mutations	38		
CNV	1		
CNA	4		
n/s	27		
nonsense + CNA	2		
***KEAP1***	**61**	**261**	**322**
mutations	40		
CNA	1		
n/s	20		
***NOTCH1***	**40**	**212**	**252**
mutations	16		
CNV	18		
n/s	6		
***NOTCH2***	**16**	**230**	**246**
mutations	13		
n/s	3		
***NOTCH3***	**10**	**163**	**173**
mutations	5		
n/s	5		
***NOTCH4***	**4**	**67**	**71**
mutations	1		
n/s	3		

CNV, copy number variation; CNA, copy number alteration; LOH, loss of heterozygosity; n/s, not specified. Bolded "+ve" = gene alteration-positive cases, bolded "-ve" = gene alteration-negative cases, bolded "total" = total number of cases for each gene.

The most common alterations were *TP53*, followed by *RB1* and *STK11* (**Table 3**). No alterations were found in *DLL3* and *ASCL1*. All alterations of *TP53*, *RB1*, *STK11* and *KEAP1* were examined in 205 cases, of which 26 cases could be classified as type I LCNEC (*TP53* and *STK11/KEAP1* alterations), and 71 cases could be classified into type II LCNECs (*TP53* and *RB1* alterations).

### Therapy for Molecular Subtypes

Chemotherapy treatment for LCNEC remains a subject of debate. In patients with advanced LCNEC, the chemotherapy regimens used in SCLC are still the standard of treatment, but results are not satisfactory (73). The type I (with *TP53* and *STK11/KEAP1* alterations) and type II LCNECs (with *TP53* and *RB1* alterations) may have a heterogeneous response to chemotherapy. Derks et al. reported patients with LCNEC tumors that carry a wild-type *RB1* gene or express the RB1 protein do better with NSCLC type chemotherapy (platinum-gemcitabine or paclitaxel) than with SCLC type chemotherapy (platinum, etoposide). In contrast, no difference was observed in LCNEC cases with the *RB1* mutation (35). Another study found that patients with NSCLC-like LCNEC treated with NSCLC-gemcitabine/taxane-platinum regimen had significantly shorter progression-free survival and overall survival than those treated with SCLC-etoposide-platinum regimen (44). It is not entirely clear why the above studies produced conflicting results. Baseline characteristics of the patients and combination therapy with irradiation could be contributing factors. Future studies in larger cohorts are needed to establish optimal protocols of chemotherapy in patients with different molecular subtypes of LCNEC.

The first antibody-drug conjugate in which DLL3 was investigated as a therapeutic target in SCLC patients is rovalpituzumab-tesirine (Rova-T) (74). Rova-T demonstrated encouraging single-agent antitumor activity with a manageable safety profile in a phase 1 trial. Unfortunately, modest clinical activity with associated toxicities led to the discontinuation of Rova-T in later studies (75). Two other novel DLL3-targeted therapies are anti-DLL3/CD3 bispecific antibodies (AMG 757) and DLL3-binding chimeric antigen receptor-modified T cells (AMG 119) (76, 77). Clinical evaluation of these therapies in SCLC patients is ongoing (NCT03319940 and NCT03392064). Since type I LCNECs show high neuroendocrine expression (ASCL1^high^/DLL3^high^/NOTCH^low^) similar to SCLC, such tumors may also be susceptible to this agent.

Recently, it was reported that the high tumor mutational burden is related to better efficacy of immunotherapy (78, 79). The tumor mutational burden is high in LCNEC (>8 mutations/Mb) and was shown to be related to PD-L1 expression (8, 9, 80). Moreover, some LCNEC cases with negative PD-L1 expression but high tumor mutation burden may respond to immunotherapy (81, 82). PD-L1 expression was reported in 10–22% of studied LCNEC cases (80, 83–85). Although PD-L1 expression is known to be distinctly higher in NSCLCs as compared to SCLCs, Hermans et al. reported PD-L1 expression was equal in *RB1* mutates (SCLC-like) and *RB1* wildtype (NSCLC-like) cases. None of the seven *STK11*-mutated samples in this study harbored PD-L1 expression. The co-occurrence of *KEAP*1 and *STK11* mutations has also been reported to be associated with poor response to immune checkpoint inhibition (13, 14, 47). Therefore, the effect of immunotherapeutic treatment might be reduced in type I LCNECs. George et al. also reported type II LCNECs exhibited a pattern of gene expression with upregulation of immune related pathways, which may impact the response of patients to immunotherapy. Clinical trials of immunotherapy in LCNEC patients are ongoing (NCT03591731, NCT02939651), but it will be necessary to consider other factors, including gene expression, in the future.

## Other Emerging Therapies

Rekhtman et al. reported that at least one alteration potentially targetable by investigational agents was present in 65% of LCNECs (30/45), being more common in *RB1* wild type LCNEC than in *RB1* mutated LCNEC (84% vs. 50%, respectively). Several case reports described a good response to EGFR-tyrosine kinase inhibitor (gefitinib/icotinib) in LCNEC with an activating *EGFR* gene mutation (exon 19) (86–88). Two case reports demonstrated that treatment with crizotinib of LCNEC with *EML4*-*ALK* rearrangement was ineffective or achieved only partial response (89, 90). On the other hand, a case of LCNEC with *PLB1*-*ALK* rearrangements reported to be sensitive to crizotinib (91). We anticipate that future studies with larger cohorts of LCNEC patients will produce more consistent results on the effect of the above targeted treatments.

The PI3K-AKT-mTOR pathway has been reported to be overactivated in lung NETs (85). Phase II clinical trials of mTOR inhibitors (everolimus) in low-to-intermediate NETs (including lung) showed encouraging results (92). Moreover, everolimus in combination with chemotherapy (carboplatin and paclitaxel) has been reported to be effective for patients with metastatic LCNEC (93). Therefore, evaluation of gene mutations in the PI3K-AKT-mTOR pathway may be useful for a targeted treatment in LCNEC.

Poly-ADP ribose polymerase (PARP) inhibitors are also being studied in combination with chemotherapy in SCLC (94). Recently, coiled-coil-domain containing 6 (CCDC6) has been indicated as a prognostic biomarker, which is also predictive of a possible response to treatment with PARP inhibitors in NSCLC (95). The CCDC6 levels are modulated by deubiquitinase ubiquitin specific protease 7 (USP7) (96). Malapelle et al. has reported that the immunostaining of pulmonary NET, including LCNEC, showed the intensity of CCDC6 staining correlated with the levels of USP7 expression (71). Moreover, the inhibition of USP7 by P5091 accelerated the degradation of CCDC6 versus control in cycloheximide treated SCLC cells *in vitro* and sensitized the cells to PARP inhibitors alone and in combination with cisplatin (71). This suggests that CCDC6 and USP7 have a predictive value for the clinical usage of USP7 inhibitors in combination with PARP inhibitors in SCLC. This can be expected to be effective in LCNEC, and further research in larger cohorts is desired in the future.

The enhancer of zeste homolog 2 (EZH2) is a histone methyltransferase that forms the polycomb repressive complex 2 (PRC2) (97). In SCLC, *EZH2* is upregulated upon inactivation of the E2F/Rb pathway and leads to aberrant methylation of its target (98). Poirier et al. reported that inhibition of *EZH2* suppressed tumor growth *in vivo* and *in vitro* in both SCLC cell lines and patient derived xenograft mouse models (99). Clinical trials of EZH2 inhibitors in SCLC patients are currently ongoing (NCT03460977). Noteworthy, high EZH2 expression in SCLC and LCNEC was reported in studies using IHC (100, 101). Therefore, EZH2 inhibition may provide new therapeutic perspectives for LCNEC as well as SCLC.

## Discussion

Although there have been remarkable scientific updates such as molecular subtyping, pathological diagnosis of LCNEC has not changed much since the release of the 2004 WHO classification (102). The diagnostic yield is limited to surgical material while small biopsies are frequently insufficient and unreliable for a definite diagnosis. However, the majority of LCNEC cases are found at the late stage of the disease progression, which makes small biopsies the common modality for tissue sampling. To improve the treatment of LCNEC, the diagnostic approach to this entity by the use of molecular classifiers or, ideally, by its cheap and robust IHC alternative has been long anticipated. There has been a large debate over whether patients with LCNEC should be treated as NSCLC or SCLC. At present, just a few clear solutions have been established through clinical trials. With this background, the recent conceptual separation of LCNEC into two different subtypes, NSCLC-like type I and SCLC-like type II, is hoped to be a promising stratification to provide better therapeutic options to patients.

In their seminal paper, Rekhtman et al. genotyped 45 LCNECs and found 56% of tumors displayed NSCLC-like molecular features, characterized by *KRAS* or *KEAP1* mutations alone or concurrently with *TP53* mutations (9). Remaining 40% of LCNECs exhibited a SCLC-like genomic profile, characterized by *RB1*/*TP53* co-alteration. Additionally, less common molecular alterations seen almost exclusively in the NSCL-like LCNEC involved the *BRAF*, *MAP2K1*, *ERBB2*, and *CDKN2A* genes, and those seen exclusively in the SCLC-like LCNEC included *MYCL1* amplification and *PTEN* mutations.

In the same study, SCLC-like LCNEC had higher Ki-67 rates and a spectrum of morphologic features closer to SCLC than NSCL-like LCNEC (9). Other groups also addressed a proliferation index detected by Ki-67 IHC for stratification of LCNEC. Milione et al. reported that LCNECs with co-mutation of *TP53* and *RB1* (SCLC-like) were significantly enriched in cases with a Ki-67 ≧55%, while the tumors with *KRAS* mutations were enriched in cases with Ki-67 <55% (39). Such findings along with molecular data suggest that there is an overlap between the two subtypes of LCNEC, and proliferation alone cannot predict genomic features.

A continuing interest in surrogate biomarkers to substitute genetic testing (i.e., in a context of LCNEC, to render molecular subtyping) is explained by the high costs of genotyping. For instance, compared to NGS, immunostaining is a simple method that can be performed in virtually any pathology laboratory. Several IHC biomarkers have been evaluated with this regard. RB1 immunoexpression was considered as a promising biomarker alternative to molecular subtyping, however, Derks et al. reported that RB1 expression was completely lost not only in almost all *RB1*-mutated LCNECs, but also in 47% of the wild-type cases (35).

The subtyping of LCNEC may be better accomplished by considering the biomarkers directly connected to the therapeutic targets. One such candidate is an expression of DLL3. Evaluating the effectiveness of DLL3 expression as a therapeutic biomarker, ideally by immunostaining, may provide a pivotal progress in the field.

Saunders et al. reported that immunohistochemical expression of the DLL3 protein was completely negative in normal lung parenchyma, but was observed in 65% of LCNEC cases (103). Several studies have reported a positive correlation between DLL3 and ASCL1 IHC expression in SCLC (21, 23). Hermans et al. has reported that DLL3 H-score and ASCL1 H-score were correlated in LCNEC (24). They demonstrated that DLL3 is highly expressed in *STK11-* and *KEAP1-*mutated type I subtype and in *TP53* wild-type tumors, as well as in tumors positive for ASCL1 and more than two neuroendocrine markers (24). Moreover, they did not find any relationship between DLL3 expression and *RB1* mutation status or Rb immunostaining. Brcic et al. analyzed high-grade pulmonary NETs and cell cultures using different DLL3 antibodies. They found no correlation between the expression of *TP53* and *RB1* and DLL3 expression in LCNEC (104).

**Figure 7:**
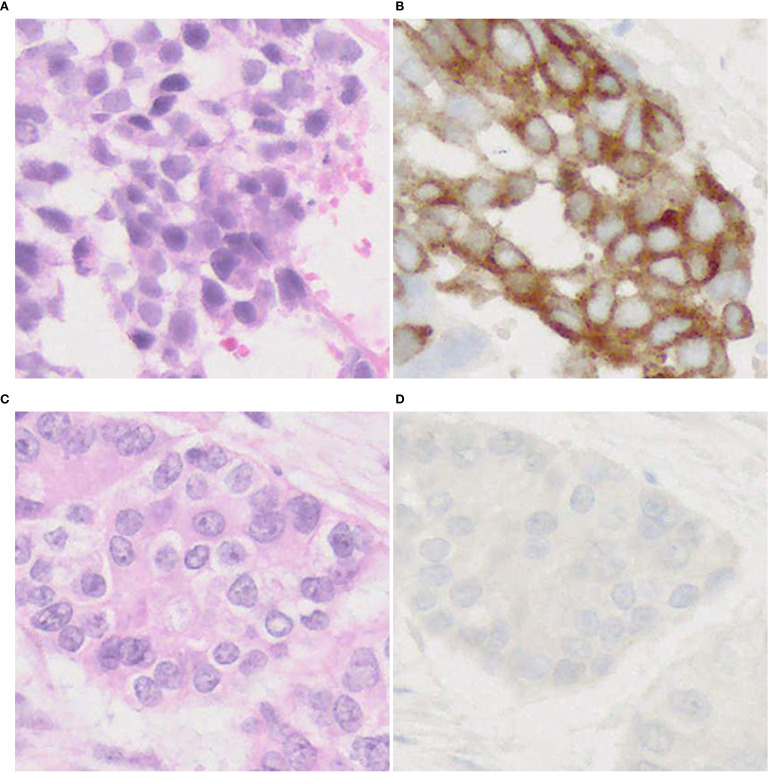
DLL3 immunophenotype of LCNEC. Representative cases with positive **(A, B)** and negative **(C, D)** expression of DLL3. Note a cytoplasmic pattern of immunostaining **(B)**. Magnification: ×40.

On DLL3 IHC staining, the majority of tumors show cytoplasmic/membranous staining (**Figure 7**), but perinuclear dot-like staining has also been reported (24). So far, it is unclear whether the pattern of staining predicts a response to DLL3-targeted therapy. These studies (24, 104) have some limitations (e.g., size of cohorts, NGS not performed in all patients, cut-off value, etc.), therefore further research is needed to verify utility of DLL3 immunostaining for adoption in practical use.

We have done a review of 205 published LCNEC cases stratified by molecular subtype limited to alterations in four genes (*TP53*, *RB1*, *STK11* and *KEAP1*). There were 108 cases (52.7%) that were not identified as either type I or type II LCNECs (**Table 4**). This suggests that the molecular spectrum of LCNEC is more heterogeneous and complex than expected.

**Table 4:** Results of a review of LCNEC molecular subtypes.

Molecular subtype	n (%)
Type I: *TP53* + *STK11* ± *KEAP1*	26 (12.7%)
Type II: *TP53* + *RB1*	71 (34.6%)
Other combinations, including:	108 (52.7%)
*RB1-, TP53-, STK11-, KEAP1-*	18
*RB1*+, *TP53* +, *STK11+, KEAP1*+	1
*RB1*+, *TP53*+, *STK11+*	6
*RB1*+, *TP53*+, *KEAP1+*	17
*STK11*+, *KEAP1+*	2
*RB1*+, *KEAP1+*	1
*RB1+*	5
*TP53+*	49
*STK11+*	6
*KEAP1+*	3
Total	205 (100%)

Carcinoid tumors have distinct epidemiology and molecular pathogenesis from LCNEC. Rekhtman et al. reported carcinoid-like LCNEC, which was characterized by MEN1 alterations and low overall mutation burden (9). Very recently, a new entity of pulmonary carcinoids named supra-carcinoids has been discovered in an integrative genomic analysis study. Supra-carcinoids appeared morphologically as atypical carcinoids, but their molecular signature corresponded to the molecular cluster of LCNEC (40, 105). In addition to sharing the molecular features of LCNEC, supra-carcinoids also showed worse prognosis, similar to survival rates in LCNEC – 10-year overall survival of 33% and 19%, respectively (vs. 59% in conventional atypical carcinoid) (105, 106). However, histological and clinical characteristics of carcinoid-like LCNEC and supra-carcinoids are yet to be defined. This suggests the possibility of molecular link between pulmonary carcinoids and LCNEC.

It is obvious that comprehensive molecular analysis in well-defined large cohorts yields additional genotypic, phenotypic, and prognostic signatures within a family of lung NETs. We may predict that new types/subtypes of LCNEC other than the recently introduced types I–II will enter the classification scheme in the future.

## Conclusion

With the rising impact of molecular pathology, the interest for reliable biomarkers grows, which can help to subclassify LCNECs and also enable personalized treatment for patients. In this review, we have discussed recent LCNEC genomic studies and treatments for LCNEC based on molecular subtype. To summarize, type I (NSCLC-like) LCNEC can be expected to respond to a DLL3 inhibitor, and type II (SCLC-like) can be expected to respond to immunotherapy. Classification of LCNECs will become important in choosing treatments. However, according to our literature review, there are many LCNECs that belong to groups other than these two categories. Moreover, there is no easy way to classify LCNEC subtypes at the clinical level. In the future, it will be necessary to study specific treatment and classification methods, and examine indicators for determining the efficacy of such methods.

## Author Contributions

MY: study design, literature review, article writing, and revision. KS: study design, literature review. AB: article writing, article revision. JF: study design, article writing, article revision. All authors contributed to the article and approved the submitted version.

## Conflict of Interest

The authors declare that the research was conducted in the absence of any commercial or financial relationships that could be construed as a potential conflict of interest.
