# Supplementary material for: Molecular Pathology of Pulmonary Large Cell Neuroendocrine Carcinoma: Novel Concepts and Treatments
Source: Front Oncol. 2021 Apr 22;11:671799. doi: 10.3389/fonc.2021.671799 (PMC8100606; doi:10.3389/fonc.2021.671799)
Supplement: Supplementary file 1 [file Table_1.docx]

Supplementary Material

**Supplemental Table 1. Frequency of gene alterations associated with LCNEC molecular subtypes**

| Reference | RB1 Alteration | Sequence RB1 | TP53  Alteration | Sequence  TP53 | Variants  TP53 | STK11  Alteration | Sequence  STK11 | Variants STK11 | KEAP1 Alteration | Sequence KEAP1 | NOTCH1 Alteration | Sequence NOTCH1 | NOTCH2 Alteration | Sequence NOTCH2 | NOTCH3 Alteration | Sequence NOTCH3 | NOTCH4 Alteration | Sequence NOTCH | DLL3 Alteration | Sequence DLL3 | ACL1 Alteration | Sequence ACL1 |
| --- | --- | --- | --- | --- | --- | --- | --- | --- | --- | --- | --- | --- | --- | --- | --- | --- | --- | --- | --- | --- | --- | --- |
| 5 | N/A |  | N/A |  |  | N/A |  |  | N/A |  | N/A |  | N/A |  | N/A |  | N/A |  | N/A |  | N/A |  |
| 5 | N/A |  | N/A |  |  | 1 | somatic alteration |  | N/A |  | N/A |  | N/A |  | N/A |  | N/A |  | N/A |  | N/A |  |
| 5 | N/A |  | N/A |  |  | 1 | somatic alteration |  | N/A |  | N/A |  | N/A |  | N/A |  | N/A |  | N/A |  | N/A |  |
| 5 | N/A |  | N/A |  |  | 1 | somatic alteration |  | N/A |  | N/A |  | N/A |  | N/A |  | N/A |  | N/A |  | N/A |  |
| 5 | N/A |  | N/A |  |  | N/A |  |  | N/A |  | N/A |  | N/A |  | N/A |  | N/A |  | N/A |  | N/A |  |
| 5 | N/A |  | N/A |  |  | 1 | somatic alteration |  | 1 | somatic alteration | N/A |  | N/A |  | N/A |  | N/A |  | N/A |  | N/A |  |
| 5 | 0 |  | N/A |  |  | 1 | somatic alteration |  | 0 |  | 0 |  | 0 |  | 0 |  | 0 |  | N/A |  | N/A |  |
| 5 | 1 | somatic alteration | N/A |  |  | 0 |  |  | 0 |  | 0 |  | 0 |  | 0 |  | 0 |  | N/A |  | N/A |  |
| 5 | 1 | somatic alteration | N/A |  |  | 0 |  |  | 0 |  | 1 | somatic alteration | 0 |  | 0 |  | 0 |  | N/A |  | N/A |  |
| 5 | 0 |  | N/A |  |  | 0 |  |  | 0 |  | 1 | somatic alteration | 0 |  | 0 |  | 0 |  | N/A |  | N/A |  |
| 5 | N/A |  | N/A |  |  | 1 | somatic alteration |  | N/A |  | N/A |  | N/A |  | N/A |  | N/A |  | N/A |  | N/A |  |
| 5 | N/A |  | N/A |  |  | N/A |  |  | N/A |  | N/A |  | N/A |  | N/A |  | N/A |  | N/A |  | N/A |  |
| 5 | 1 | somatic alteration | N/A |  |  | 0 |  |  | 0 |  | 1 | somatic alteration | 0 |  | 0 |  | 0 |  | N/A |  | N/A |  |
| 5 | N/A |  | N/A |  |  | N/A |  |  | N/A |  | N/A |  | N/A |  | N/A |  | N/A |  | N/A |  | N/A |  |
| 5 | 0 |  | N/A |  |  | 1 | somatic alteration |  | 0 |  | 0 |  | 0 |  | 0 |  | 0 |  | N/A |  | N/A |  |
| 5 | 1 | somatic alteration | N/A |  |  | 0 |  |  | 0 |  | 1 | somatic alteration | 0 |  | 0 |  | 0 |  | N/A |  | N/A |  |
| 5 | N/A |  | N/A |  |  | N/A |  |  | N/A |  | N/A |  | N/A |  | N/A |  | N/A |  | N/A |  | N/A |  |
| 5 | 0 |  | N/A |  |  | 1 | somatic alteration |  | 0 |  | 0 |  | 0 |  | 0 |  | 0 |  | N/A |  | N/A |  |
| 5 | N/A |  | N/A |  |  | 0 |  |  | 0 |  | N/A |  | N/A |  | N/A |  | N/A |  | N/A |  | N/A |  |
| 5 | 1 | somatic alteration | N/A |  |  | 0 |  |  | 0 |  | 0 |  | 0 |  | 0 |  | 0 |  | N/A |  | N/A |  |
| 5 | 0 |  | N/A |  |  | 0 |  |  | 1 | somatic alteration | 0 |  | 0 |  | 0 |  | 0 |  | N/A |  | N/A |  |
| 5 | N/A |  | N/A |  |  | N/A |  |  | N/A |  | N/A |  | N/A |  | N/A |  | N/A |  | N/A |  | N/A |  |
| 5 | 0 |  | N/A |  |  | 1 | somatic alteration |  | 0 |  | 0 |  | 0 |  | 0 |  | 0 |  | N/A |  | N/A |  |
| 5 | 0 |  | N/A |  |  | 0 |  |  | 0 |  | 0 |  | 0 |  | 0 |  | 0 |  | N/A |  | N/A |  |
| 5 | 1 | somatic alteration | N/A |  |  | 0 |  |  | 0 |  | 0 |  | 0 |  | 0 |  | 0 |  | N/A |  | N/A |  |
| 5 | 0 |  | N/A |  |  | 1 | somatic alteration |  | 0 |  | 0 |  | 0 |  | 0 |  | 0 |  | N/A |  | N/A |  |
| 5 | 1 | somatic alteration | N/A |  |  | 0 |  |  | 0 |  | 0 |  | 0 |  | 0 |  | 0 |  | N/A |  | N/A |  |
| 5 | 0 |  | N/A |  |  | 1 | somatic alteration |  | 0 |  | 0 |  | 0 |  | 0 |  | 0 |  | N/A |  | N/A |  |
| 5 | 1 | somatic alteration | N/A |  |  | 0 |  |  | 0 |  | 0 |  | 0 |  | 0 |  | 0 |  | N/A |  | N/A |  |
| 5 | 0 |  | N/A |  |  | 0 |  |  | 0 |  | 0 |  | 0 |  | 0 |  | 0 |  | N/A |  | N/A |  |
| 5 | 0 |  | N/A |  |  | 1 | somatic alteration |  | 0 |  | 0 |  | 0 |  | 0 |  | 1 | somatic alteration | N/A |  | N/A |  |
| 5 | N/A |  | N/A |  |  | N/A |  |  | N/A |  | N/A |  | N/A |  | N/A |  | N/A |  | N/A |  | N/A |  |
| 5 | 1 | somatic alteration | N/A |  |  | 0 |  |  | 0 |  | 0 |  | 0 |  | 0 |  | 0 |  | N/A |  | N/A |  |
| 5 | 1 | somatic alteration | N/A |  |  | 0 |  |  | 0 |  | 0 |  | 1 | somatic alteration | 0 |  | 0 |  | N/A |  | N/A |  |
| 5 | 0 |  | N/A |  |  | 0 |  |  | 0 |  | 0 |  | 0 |  | 0 |  | 0 |  | N/A |  | N/A |  |
| 5 | N/A |  | N/A |  |  | N/A |  |  | 1 | somatic alteration | N/A |  | N/A |  | N/A |  | N/A |  | N/A |  | N/A |  |
| 5 | 0 |  | N/A |  |  | 0 |  |  | 0 |  | 0 |  | 0 |  | 1 | somatic alteration | 0 |  | N/A |  | N/A |  |
| 5 | 1 | somatic alteration | N/A |  |  | 0 |  |  | 0 |  | 0 |  | 0 |  | 0 |  | 0 |  | N/A |  | N/A |  |
| 5 | 0 |  | N/A |  |  | 1 | somatic alteration |  | 0 |  | 0 |  | 0 |  | 0 |  | 0 |  | N/A |  | N/A |  |
| 5 | 1 | somatic alteration | N/A |  |  | 0 |  |  | 0 |  | 0 |  | 0 |  | 0 |  | 0 |  | N/A |  | N/A |  |
| 5 | 1 | somatic alteration | N/A |  |  | 0 |  |  | 0 |  | 0 |  | 0 |  | 0 |  | 0 |  | N/A |  | N/A |  |
| 5 | 1 | somatic alteration | N/A |  |  | 0 |  |  | 0 |  | 0 |  | 0 |  | 1 | somatic alteration | 0 |  | N/A |  | N/A |  |
| 5 | 0 |  | N/A |  |  | 1 | somatic alteration |  | 1 | somatic alteration | 0 |  | 0 |  | 0 |  | 0 |  | N/A |  | N/A |  |
| 5 | 0 |  | N/A |  |  | 1 | somatic alteration |  | 1 | somatic alteration | 0 |  | 0 |  | 0 |  | 1 | somatic alteration | N/A |  | N/A |  |
| 5 | 0 |  | N/A |  |  | 0 |  |  | 1 | somatic alteration | 0 |  | 0 |  | 0 |  | 0 |  | N/A |  | N/A |  |
| 5 | 0 |  | N/A |  |  | 0 |  |  | 1 | somatic alteration | 0 |  | 1 | somatic alteration | 1 | somatic alteration | 0 |  | N/A |  | N/A |  |
| 5 | 0 |  | N/A |  |  | 0 |  |  | 1 | somatic alteration | 0 |  | 0 |  | 0 |  | 0 |  | N/A |  | N/A |  |
| 5 | 0 |  | N/A |  |  | 0 |  |  | 1 | somatic alteration | 0 |  | 0 |  | 0 |  | 0 |  | N/A |  | N/A |  |
| 5 | 1 | somatic alteration | N/A |  |  | 0 |  |  | 1 | somatic alteration | 0 |  | 0 |  | 0 |  | 0 |  | N/A |  | N/A |  |
| 5 | 0 |  | N/A |  |  | 1 | somatic alteration |  | 0 |  | 0 |  | 0 |  | 0 |  | 0 |  | N/A |  | N/A |  |
| 5 | 1 | somatic alteration | N/A |  |  | 0 |  |  | 0 |  | 0 |  | 0 |  | 0 |  | 0 |  | N/A |  | N/A |  |
| 5 | 0 |  | N/A |  |  | 0 |  |  | 1 | somatic alteration | 0 |  | 0 |  | 0 |  | 0 |  | N/A |  | N/A |  |
| 5 | 0 |  | N/A |  |  | 1 | somatic alteration |  | 0 |  | 0 |  | 0 |  | 0 |  | 1 | somatic alteration | N/A |  | N/A |  |
| 5 | 0 |  | N/A |  |  | 0 |  |  | 0 |  | 1 | somatic alteration | 0 |  | 0 |  | 0 |  | N/A |  | N/A |  |
| 5 | 1 | somatic alteration | N/A |  |  | 0 |  |  | 0 |  | 0 |  | 0 |  | 0 |  | 0 |  | N/A |  | N/A |  |
| 5 | 1 | somatic alteration | N/A |  |  | 0 |  |  | 0 |  | 0 |  | 0 |  | 0 |  | 0 |  | N/A |  | N/A |  |
| 5 | 1 | somatic alteration | N/A |  |  | 1 | somatic alteration |  | 0 |  | 0 |  | 0 |  | 0 |  | 0 |  | N/A |  | N/A |  |
| 5 | 0 |  | N/A |  |  | 0 |  |  | 0 |  | 0 |  | 0 |  | 0 |  | 0 |  | N/A |  | N/A |  |
| 5 | 0 |  | N/A |  |  | 0 |  |  | 1 | somatic alteration | 0 |  | 0 |  | 0 |  | 0 |  | N/A |  | N/A |  |
| 5 | 1 | somatic alteration | N/A |  |  | 0 |  |  | 1 | somatic alteration | 0 |  | 0 |  | 0 |  | 0 |  | N/A |  | N/A |  |
| 5 | 0 |  | N/A |  |  | 1 | somatic alteration |  | 0 |  | 0 |  | 0 |  | 0 |  | 0 |  | N/A |  | N/A |  |
| 5 | 0 |  | N/A |  |  | 0 |  |  | 0 |  | 0 |  | 0 |  | 0 |  | 0 |  | N/A |  | N/A |  |
| 5 | 0 |  | N/A |  |  | 0 |  |  | 0 |  | 0 |  | 0 |  | 0 |  | 0 |  | N/A |  | N/A |  |
| 5 | 0 |  | N/A |  |  | 0 |  |  | 0 |  | 1 | somatic alteration | 0 |  | 0 |  | 0 |  | N/A |  | N/A |  |
| 5 | 0 |  | N/A |  |  | 1 | somatic alteration |  | 0 |  | 0 |  | 0 |  | 0 |  | 0 |  | N/A |  | N/A |  |
| 5 | 0 |  | N/A |  |  | 1 | somatic alteration |  | 1 | somatic alteration | 0 |  | 0 |  | 0 |  | 0 |  | N/A |  | N/A |  |
| 7 | 1 | truncating | 1 | missense |  | 0 |  |  | 0 |  | N/A |  | N/A |  | N/A |  | N/A |  | N/A |  | N/A |  |
| 7 | 1 | truncating | 1 | truncating |  | 0 |  |  | 0 |  | N/A |  | N/A |  | N/A |  | N/A |  | N/A |  | N/A |  |
| 7 | 1 | CAN loss | 1 | missense |  | 0 |  |  | 0 |  | N/A |  | N/A |  | N/A |  | N/A |  | N/A |  | N/A |  |
| 7 | 1 | CAN loss | 1 | missense |  | 0 |  |  | 0 |  | N/A |  | N/A |  | N/A |  | N/A |  | N/A |  | N/A |  |
| 7 | 1 | missense | 1 | truncating |  | 0 |  |  | 0 |  | N/A |  | N/A |  | N/A |  | N/A |  | N/A |  | N/A |  |
| 7 | 1 | truncating | 1 | truncating |  | 0 |  |  | 0 |  | N/A |  | N/A |  | N/A |  | N/A |  | N/A |  | N/A |  |
| 7 | 1 | truncating | 1 | missense |  | 0 |  |  | 0 |  | N/A |  | N/A |  | N/A |  | N/A |  | N/A |  | N/A |  |
| 7 | 1 | CAN loss | 1 | missense |  | 0 |  |  | 0 |  | N/A |  | N/A |  | N/A |  | N/A |  | N/A |  | N/A |  |
| 7 | 1 | truncating | 1 | truncating |  | 0 |  |  | 0 |  | N/A |  | N/A |  | N/A |  | N/A |  | N/A |  | N/A |  |
| 7 | 0 |  | 1 | missense |  | 0 |  |  | 0 |  | N/A |  | N/A |  | N/A |  | N/A |  | N/A |  | N/A |  |
| 7 | 1 | missense | 1 | missense |  | 0 |  |  | 0 |  | N/A |  | N/A |  | N/A |  | N/A |  | N/A |  | N/A |  |
| 7 | 0 |  | 1 | missense |  | 0 |  |  | 1 | missense | N/A |  | N/A |  | N/A |  | N/A |  | N/A |  | N/A |  |
| 7 | 1 | truncating | 1 | truncating |  | 0 |  |  | 1 | missense | N/A |  | N/A |  | N/A |  | N/A |  | N/A |  | N/A |  |
| 7 | 1 | truncating | 1 | missense |  | 0 |  |  | 1 | missense | N/A |  | N/A |  | N/A |  | N/A |  | N/A |  | N/A |  |
| 7 | 1 | truncating | 1 | truncating |  | 0 |  |  | 1 | missense | N/A |  | N/A |  | N/A |  | N/A |  | N/A |  | N/A |  |
| 7 | 1 | truncating | 1 | missense |  | 0 |  |  | 1 | missense | N/A |  | N/A |  | N/A |  | N/A |  | N/A |  | N/A |  |
| 7 | 1 | truncating | 1 | missense |  | 0 |  |  | 1 | CAN loss | N/A |  | N/A |  | N/A |  | N/A |  | N/A |  | N/A |  |
| 7 | 1 | truncating | 1 | truncating |  | 0 |  |  | 0 |  | N/A |  | N/A |  | N/A |  | N/A |  | N/A |  | N/A |  |
| 7 | 0 |  | 1 | truncating |  | 1 | truncating |  | 1 | missense | N/A |  | N/A |  | N/A |  | N/A |  | N/A |  | N/A |  |
| 7 | 0 |  | 1 | truncating |  | 1 | truncating |  | 0 |  | N/A |  | N/A |  | N/A |  | N/A |  | N/A |  | N/A |  |
| 7 | 0 |  | 1 | missense |  | 1 | missense |  | 0 |  | N/A |  | N/A |  | N/A |  | N/A |  | N/A |  | N/A |  |
| 7 | 0 |  | 0 |  |  | 1 | missense |  | 0 |  | N/A |  | N/A |  | N/A |  | N/A |  | N/A |  | N/A |  |
| 7 | 0 |  | 0 |  |  | 1 | missense |  | 0 |  | N/A |  | N/A |  | N/A |  | N/A |  | N/A |  | N/A |  |
| 7 | 0 |  | 0 |  |  | 0 |  |  | 1 | truncating | N/A |  | N/A |  | N/A |  | N/A |  | N/A |  | N/A |  |
| 7 | 1 | truncating | 0 |  |  | 0 |  |  | 0 |  | N/A |  | N/A |  | N/A |  | N/A |  | N/A |  | N/A |  |
| 7 | 0 |  | 1 | missense |  | 0 |  |  | 0 |  | N/A |  | N/A |  | N/A |  | N/A |  | N/A |  | N/A |  |
| 7 | 0 |  | 1 | truncating |  | 0 |  |  | 0 |  | N/A |  | N/A |  | N/A |  | N/A |  | N/A |  | N/A |  |
| 7 | 0 |  | 1 | missense |  | 0 |  |  | 0 |  | N/A |  | N/A |  | N/A |  | N/A |  | N/A |  | N/A |  |
| 7 | 0 |  | 1 | truncating |  | 1 | missense |  | 1 | missense | N/A |  | N/A |  | N/A |  | N/A |  | N/A |  | N/A |  |
| 7 | 0 |  | 1 | truncating |  | 1 | truncating |  | 1 | truncating | N/A |  | N/A |  | N/A |  | N/A |  | N/A |  | N/A |  |
| 7 | 0 |  | 1 | missense |  | 1 | truncating |  | 1 | truncating | N/A |  | N/A |  | N/A |  | N/A |  | N/A |  | N/A |  |
| 7 | 0 |  | 1 | missense |  | 1 | truncating |  | 0 |  | N/A |  | N/A |  | N/A |  | N/A |  | N/A |  | N/A |  |
| 7 | 0 |  | 1 | missense |  | 1 | truncating |  | 0 |  | N/A |  | N/A |  | N/A |  | N/A |  | N/A |  | N/A |  |
| 7 | 0 |  | 0 |  |  | 1 | missense |  | 0 |  | N/A |  | N/A |  | N/A |  | N/A |  | N/A |  | N/A |  |
| 7 | 0 |  | 1 | missense |  | 1 | missense |  | 0 |  | N/A |  | N/A |  | N/A |  | N/A |  | N/A |  | N/A |  |
| 7 | 0 |  | 0 |  |  | 1 | truncating |  | 0 |  | N/A |  | N/A |  | N/A |  | N/A |  | N/A |  | N/A |  |
| 7 | 0 |  | 0 |  |  | 1 | truncating |  | 0 |  | N/A |  | N/A |  | N/A |  | N/A |  | N/A |  | N/A |  |
| 7 | 0 |  | 1 | truncating |  | 1 | truncating |  | 0 |  | N/A |  | N/A |  | N/A |  | N/A |  | N/A |  | N/A |  |
| 7 | 0 |  | 1 | missense |  | 0 |  |  | 1 | missense | N/A |  | N/A |  | N/A |  | N/A |  | N/A |  | N/A |  |
| 7 | 0 |  | 1 | missense |  | 0 |  |  | 1 | truncating | N/A |  | N/A |  | N/A |  | N/A |  | N/A |  | N/A |  |
| 7 | 0 |  | 1 | missense |  | 0 |  |  | 1 | missense | N/A |  | N/A |  | N/A |  | N/A |  | N/A |  | N/A |  |
| 7 | 0 |  | 0 |  |  | 0 |  |  | 0 |  | N/A |  | N/A |  | N/A |  | N/A |  | N/A |  | N/A |  |
| 7 | 0 |  | 1 | truncating |  | 0 |  |  | 0 |  | N/A |  | N/A |  | N/A |  | N/A |  | N/A |  | N/A |  |
| 7 | 0 |  | 0 |  |  | 0 |  |  | 0 |  | N/A |  | N/A |  | N/A |  | N/A |  | N/A |  | N/A |  |
| 7 | 0 |  | 0 |  |  | 0 |  |  | 0 |  | N/A |  | N/A |  | N/A |  | N/A |  | N/A |  | N/A |  |
| 27 | N/A |  | 1 | missense |  | 1 | missense |  | N/A |  | N/A |  | N/A |  | N/A |  | N/A | N/A | N/A |  | N/A |  |
| 27 | N/A |  | 1 | other mutation, CAN gain |  | 1 | frame sift |  | N/A |  | N/A |  | N/A |  | N/A |  | N/A | N/A | N/A |  | N/A |  |
| 27 | N/A |  | 1 | nonsense |  | 1 | nonsense |  | N/A |  | N/A |  | N/A |  | N/A |  | N/A | N/A | N/A |  | N/A |  |
| 27 | N/A |  | 1 | nonsense |  | 0 |  |  | N/A |  | N/A |  | N/A |  | N/A |  | N/A | N/A | N/A |  | N/A |  |
| 27 | N/A |  | 1 | splice, CAN loss |  | 0 |  |  | N/A |  | N/A |  | N/A |  | N/A |  | N/A | N/A | N/A |  | N/A |  |
| 27 | N/A |  | 1 | nonsense |  | 0 |  |  | N/A |  | N/A |  | N/A |  | N/A |  | N/A | N/A | N/A |  | N/A |  |
| 27 | N/A |  | 1 | missense, CAN loss |  | 0 |  |  | N/A |  | N/A |  | N/A |  | N/A |  | N/A | N/A | N/A |  | N/A |  |
| 27 | N/A |  | 1 | missense |  | 0 |  |  | N/A |  | N/A |  | N/A |  | N/A |  | N/A | N/A | N/A |  | N/A |  |
| 27 | N/A |  | 1 | missense |  | 0 |  |  | N/A |  | N/A |  | N/A |  | N/A |  | N/A | N/A | N/A |  | N/A |  |
| 27 | N/A |  | 1 | nonsense |  | 0 |  |  | N/A |  | N/A |  | N/A |  | N/A |  | N/A | N/A | N/A |  | N/A |  |
| 27 | N/A |  | 1 | frame shift InsDel,CAN loss |  | 1 | CAN loss |  | N/A |  | N/A |  | N/A |  | N/A |  | N/A | N/A | N/A |  | N/A |  |
| 27 | N/A |  | 1 | missense, CAN loss |  | 1 | CAN gain |  | N/A |  | N/A |  | N/A |  | N/A |  | N/A | N/A | N/A |  | N/A |  |
| 27 | N/A |  | 1 | missense |  | 0 |  |  | N/A |  | N/A |  | N/A |  | N/A |  | N/A | N/A | N/A |  | N/A |  |
| 27 | N/A |  | 1 | missense, CAN loss |  | 0 |  |  | N/A |  | N/A |  | N/A |  | N/A |  | N/A | N/A | N/A |  | N/A |  |
| 27 | N/A |  | 1 | missense, CAN loss |  | 0 |  |  | N/A |  | N/A |  | N/A |  | N/A |  | N/A | N/A | N/A |  | N/A |  |
| 27 | N/A |  | 1 | missense |  | 0 |  |  | N/A |  | N/A |  | N/A |  | N/A |  | N/A | N/A | N/A |  | N/A |  |
| 27 | N/A |  | 1 | missense, CAN loss |  | 1 | CAN loss |  | N/A |  | N/A |  | N/A |  | N/A |  | N/A | N/A | N/A |  | N/A |  |
| 27 | N/A |  | 1 | missense, CAN loss |  | 0 |  |  | N/A |  | N/A |  | N/A |  | N/A |  | N/A | N/A | N/A |  | N/A |  |
| 27 | N/A |  | 1 | missense |  | 0 |  |  | N/A |  | N/A |  | N/A |  | N/A |  | N/A | N/A | N/A |  | N/A |  |
| 27 | N/A |  | 1 | missense |  | 0 |  |  | N/A |  | N/A |  | N/A |  | N/A |  | N/A | N/A | N/A |  | N/A |  |
| 27 | N/A |  | 1 | missense |  | 0 |  |  | N/A |  | N/A |  | N/A |  | N/A |  | N/A | N/A | N/A |  | N/A |  |
| 27 | N/A |  | 1 | missense |  | 0 |  |  | N/A |  | N/A |  | N/A |  | N/A |  | N/A | N/A | N/A |  | N/A |  |
| 27 | N/A |  | 1 | splice, CAN loss |  | 0 |  |  | N/A |  | N/A |  | N/A |  | N/A |  | N/A | N/A | N/A |  | N/A |  |
| 27 | N/A |  | 1 | missense |  | 0 |  |  | N/A |  | N/A |  | N/A |  | N/A |  | N/A | N/A | N/A |  | N/A |  |
| 27 | N/A |  | 1 | missense |  | 1 | CAN loss |  | N/A |  | N/A |  | N/A |  | N/A |  | N/A | N/A | N/A |  | N/A |  |
| 27 | N/A |  | 1 | nonsense |  | 0 |  |  | N/A |  | N/A |  | N/A |  | N/A |  | N/A | N/A | N/A |  | N/A |  |
| 27 | N/A |  | 1 | frame shift InsDel |  | 0 |  |  | N/A |  | N/A |  | N/A |  | N/A |  | N/A | N/A | N/A |  | N/A |  |
| 27 | N/A |  | 1 | missense |  | 0 |  |  | N/A |  | N/A |  | N/A |  | N/A |  | N/A | N/A | N/A |  | N/A |  |
| 27 | N/A |  | 1 | CAN loss |  | 1 | nonsense, CAN loss |  | N/A |  | N/A |  | N/A |  | N/A |  | N/A | N/A | N/A |  | N/A |  |
| 27 | N/A |  | 1 | CAN loss |  | 1 | nonsense, CAN loss |  | N/A |  | N/A |  | N/A |  | N/A |  | N/A | N/A | N/A |  | N/A |  |
| 27 | N/A |  | 0 |  |  | 0 |  |  | N/A |  | N/A |  | N/A |  | N/A |  | N/A | N/A | N/A |  | N/A |  |
| 27 | N/A |  | 0 |  |  | 0 |  |  | N/A |  | N/A |  | N/A |  | N/A |  | N/A | N/A | N/A |  | N/A |  |
| 28 | 1 | indel | 1 | nonsynonymous |  | N/A |  |  | N/A |  | 0 |  | 0 |  | 0 |  | N/A |  | N/A |  | N/A |  |
| 28 | 1 | indel | 1 | nonsynonymous |  | N/A |  |  | N/A |  | 0 |  | 0 |  | 0 |  | N/A |  | N/A |  | N/A |  |
| 28 | 0 |  | 1 | nonsynonymous |  | N/A |  |  | N/A |  | 0 |  | 0 |  | 0 |  | N/A |  | N/A |  | N/A |  |
| 28 | 0 |  | 0 |  |  | N/A |  |  | N/A |  | 0 |  | 0 |  | 0 |  | N/A |  | N/A |  | N/A |  |
| 28 | 1 | indel | 1 | nonsynonymous |  | N/A |  |  | N/A |  | 0 |  | 0 |  | 0 |  | N/A |  | N/A |  | N/A |  |
| 28 | 0 |  | 0 |  |  | N/A |  |  | N/A |  | 0 |  | 0 |  | 0 |  | N/A |  | N/A |  | N/A |  |
| 28 | 0 |  | 1 | nonsynonymous |  | N/A |  |  | N/A |  | 0 |  | 0 |  | 0 |  | N/A |  | N/A |  | N/A |  |
| 28 | 1 | nonsynonymous | 0 |  |  | N/A |  |  | N/A |  | 0 |  | 0 |  | 0 |  | N/A |  | N/A |  | N/A |  |
| 28 | 1 | nonsynonymous | 1 | nonsynonymous |  | N/A |  |  | N/A |  | 0 |  | 0 |  | 0 |  | N/A |  | N/A |  | N/A |  |
| 28 | 0 |  | 1 | nonsynonymous |  | N/A |  |  | N/A |  | 0 |  | 0 |  | 0 |  | N/A |  | N/A |  | N/A |  |
| 28 | 0 |  | 1 | nonsynonymous |  | N/A |  |  | N/A |  | 0 |  | 0 |  | 0 |  | N/A |  | N/A |  | N/A |  |
| 28 | 1 | indel | 1 | nonsynonymous |  | N/A |  |  | N/A |  | 0 |  | 0 |  | 0 |  | N/A |  | N/A |  | N/A |  |
| 28 | 0 |  | 1 | nonsynonymous |  | N/A |  |  | N/A |  | 0 |  | 0 |  | 0 |  | N/A |  | N/A |  | N/A |  |
| 28 | 0 |  | 0 |  |  | N/A |  |  | N/A |  | 0 |  | 0 |  | 0 |  | N/A |  | N/A |  | N/A |  |
| 28 | 0 |  | 0 |  |  | N/A |  |  | N/A |  | 1 | nonsynonymous | 0 |  | 0 |  | N/A |  | N/A |  | N/A |  |
| 28 | 0 |  | 0 |  |  | N/A |  |  | N/A |  | 0 |  | 0 |  | 0 |  | N/A |  | N/A |  | N/A |  |
| 28 | 0 |  | 1 | nonsynonymous |  | N/A |  |  | N/A |  | 0 |  | 0 |  | 0 |  | N/A |  | N/A |  | N/A |  |
| 28 | 0 |  | 1 | nonsynonymous |  | N/A |  |  | N/A |  | 0 |  | 1 | nonsynonymous | 0 |  | N/A |  | N/A |  | N/A |  |
| 28 | 0 |  | 1 | nonsynonymous |  | N/A |  |  | N/A |  | 1 | nonsynonymous, indel | 0 |  | 0 |  | N/A |  | N/A |  | N/A |  |
| 28 | 1 | indel | 0 |  |  | N/A |  |  | N/A |  | 0 |  | 0 |  | 0 |  | N/A |  | N/A |  | N/A |  |
| 28 | 0 |  | 1 | nonsynonymous |  | N/A |  |  | N/A |  | 0 |  | 0 |  | 0 |  | N/A |  | N/A |  | N/A |  |
| 28 | 0 |  | 0 |  |  | N/A |  |  | N/A |  | 0 |  | 0 |  | 0 |  | N/A |  | N/A |  | N/A |  |
| 28 | 0 |  | 0 |  |  | N/A |  |  | N/A |  | 0 |  | 0 |  | 0 |  | N/A |  | N/A |  | N/A |  |
| 28 | 1 | indel | 0 |  |  | N/A |  |  | N/A |  | 0 |  | 0 |  | 0 |  | N/A |  | N/A |  | N/A |  |
| 28 | 1 | nonsynonymous | 1 | nonsynonymous |  | N/A |  |  | N/A |  | 0 |  | 0 |  | 0 |  | N/A |  | N/A |  | N/A |  |
| 28 | 1 | indel | 1 | nonsynonymous |  | N/A |  |  | N/A |  | 0 |  | 0 |  | 0 |  | N/A |  | N/A |  | N/A |  |
| 28 | 0 |  | 1 | indel |  | N/A |  |  | N/A |  | 0 |  | 0 |  | 0 |  | N/A |  | N/A |  | N/A |  |
| 28 | 0 |  | 1 | nonsynonymous |  | N/A |  |  | N/A |  | 0 |  | 0 |  | 0 |  | N/A |  | N/A |  | N/A |  |
| 28 | 0 |  | 0 |  |  | N/A |  |  | N/A |  | 0 |  | 0 |  | 0 |  | N/A |  | N/A |  | N/A |  |
| 28 | 0 |  | 1 | nonsynonymous |  | N/A |  |  | N/A |  | 0 |  | 0 |  | 0 |  | N/A |  | N/A |  | N/A |  |
| 28 | 0 |  | 0 |  |  | N/A |  |  | N/A |  | 0 |  | 0 |  | 0 |  | N/A |  | N/A |  | N/A |  |
| 28 | 0 |  | 0 |  |  | N/A |  |  | N/A |  | 0 |  | 0 |  | 0 |  | N/A |  | N/A |  | N/A |  |
| 28 | 0 |  | 1 | nonsynonymous |  | N/A |  |  | N/A |  | 0 |  | 0 |  | 0 |  | N/A |  | N/A |  | N/A |  |
| 28 | 0 |  | 1 | nonsynonymous |  | N/A |  |  | N/A |  | 0 |  | 0 |  | 1 | nonsynonymous | N/A |  | N/A |  | N/A |  |
| 28 | 0 |  | 1 | nonsynonymous |  | N/A |  |  | N/A |  | 1 | nonsynonymous | 0 |  | 0 |  | N/A |  | N/A |  | N/A |  |
| 28 | 0 |  | 1 | nonsynonymous |  | N/A |  |  | N/A |  | 0 |  | 0 |  | 0 |  | N/A |  | N/A |  | N/A |  |
| 28 | 1 | nonsynonymous | 0 |  |  | N/A |  |  | N/A |  | 0 |  | 0 |  | 0 |  | N/A |  | N/A |  | N/A |  |
| 28 | 0 |  | 1 | nonsynonymous |  | N/A |  |  | N/A |  | 0 |  | 0 |  | 0 |  | N/A |  | N/A |  | N/A |  |
| 28 | 0 |  | 1 | nonsynonymous |  | N/A |  |  | N/A |  | 0 |  | 0 |  | 0 |  | N/A |  | N/A |  | N/A |  |
| 28 | 0 |  | 1 | nonsynonymous |  | N/A |  |  | N/A |  | 0 |  | 0 |  | 0 |  | N/A |  | N/A |  | N/A |  |
| 28 | 1 | nonsynonymous | 1 | nonsynonymous |  | N/A |  |  | N/A |  | 0 |  | 0 |  | 0 |  | N/A |  | N/A |  | N/A |  |
| 28 | 0 |  | 1 | nonsynonymous |  | N/A |  |  | N/A |  | 1 | indel | 0 |  | 0 |  | N/A |  | N/A |  | N/A |  |
| 28 | 0 |  | 1 | nonsynonymous |  | N/A |  |  | N/A |  | 0 |  | 0 |  | 0 |  | N/A |  | N/A |  | N/A |  |
| 28 | 0 |  | 1 | nonsynonymous |  | N/A |  |  | N/A |  | 0 |  | 0 |  | 0 |  | N/A |  | N/A |  | N/A |  |
| 28 | 0 |  | 1 | indel |  | N/A |  |  | N/A |  | 0 |  | 0 |  | 0 |  | N/A |  | N/A |  | N/A |  |
| 28 | 0 |  | 0 |  |  | N/A |  |  | N/A |  | 0 |  | 0 |  | 0 |  | N/A |  | N/A |  | N/A |  |
| 28 | 0 |  | 1 | nonsynonymous |  | N/A |  |  | N/A |  | 0 |  | 0 |  | 0 |  | N/A |  | N/A |  | N/A |  |
| 28 | 0 |  | 1 | nonsynonymous |  | N/A |  |  | N/A |  | 1 | indel | 0 |  | 0 |  | N/A |  | N/A |  | N/A |  |
| 28 | 0 |  | 1 | nonsynonymous |  | N/A |  |  | N/A |  | 0 |  | 0 |  | 0 |  | N/A |  | N/A |  | N/A |  |
| 28 | 0 |  | 1 | nonsynonymous |  | N/A |  |  | N/A |  | 0 |  | 0 |  | 0 |  | N/A |  | N/A |  | N/A |  |
| 28 | 0 |  | 0 |  |  | N/A |  |  | N/A |  | 0 |  | 0 |  | 0 |  | N/A |  | N/A |  | N/A |  |
| 28 | 0 |  | 0 |  |  | N/A |  |  | N/A |  | 0 |  | 0 |  | 0 |  | N/A |  | N/A |  | N/A |  |
| 28 | 0 |  | 1 | nonsynonymous |  | N/A |  |  | N/A |  | 0 |  | 0 |  | 0 |  | N/A |  | N/A |  | N/A |  |
| 28 | 0 |  | 1 | nonsynonymous |  | N/A |  |  | N/A |  | 0 |  | 0 |  | 0 |  | N/A |  | N/A |  | N/A |  |
| 28 | 0 |  | 1 | nonsynonymous |  | N/A |  |  | N/A |  | 0 |  | 0 |  | 0 |  | N/A |  | N/A |  | N/A |  |
| 28 | 0 |  | 1 | nonsynonymous |  | N/A |  |  | N/A |  | 0 |  | 0 |  | 0 |  | N/A |  | N/A |  | N/A |  |
| 28 | 0 |  | 1 | nonsynonymous |  | N/A |  |  | N/A |  | 0 |  | 0 |  | 0 |  | N/A |  | N/A |  | N/A |  |
| 28 | 0 |  | 1 | nonsynonymous |  | N/A |  |  | N/A |  | 0 |  | 0 |  | 0 |  | N/A |  | N/A |  | N/A |  |
| 28 | 0 |  | 1 | nonsynonymous |  | N/A |  |  | N/A |  | 0 |  | 0 |  | 0 |  | N/A |  | N/A |  | N/A |  |
| 28 | 1 | nonsynonymous | 0 |  |  | N/A |  |  | N/A |  | 0 |  | 0 |  | 0 |  | N/A |  | N/A |  | N/A |  |
| 28 | 0 |  | 0 |  |  | N/A |  |  | N/A |  | 0 |  | 0 |  | 0 |  | N/A |  | N/A |  | N/A |  |
| 28 | 0 |  | 1 | nonsynonymous |  | N/A |  |  | N/A |  | 0 |  | 0 |  | 0 |  | N/A |  | N/A |  | N/A |  |
| 28 | 0 |  | 1 | nonsynonymous |  | N/A |  |  | N/A |  | 0 |  | 0 |  | 0 |  | N/A |  | N/A |  | N/A |  |
| 28 | 0 |  | 1 | nonsynonymous |  | N/A |  |  | N/A |  | 0 |  | 0 |  | 0 |  | N/A |  | N/A |  | N/A |  |
| 28 | 0 |  | 1 | nonsynonymous |  | N/A |  |  | N/A |  | 0 |  | 0 |  | 0 |  | N/A |  | N/A |  | N/A |  |
| 28 | 1 | nonsynonymous | 0 |  |  | N/A |  |  | N/A |  | 0 |  | 0 |  | 0 |  | N/A |  | N/A |  | N/A |  |
| 28 | 1 | nonsynonymous | 0 |  |  | N/A |  |  | N/A |  | 0 |  | 0 |  | 0 |  | N/A |  | N/A |  | N/A |  |
| 28 | 0 |  | 1 | nonsynonymous |  | N/A |  |  | N/A |  | 0 |  | 0 |  | 0 |  | N/A |  | N/A |  | N/A |  |
| 28 | 0 |  | 1 | nonsynonymous |  | N/A |  |  | N/A |  | 0 |  | 0 |  | 0 |  | N/A |  | N/A |  | N/A |  |
| 28 | 0 |  | 1 | nonsynonymous |  | N/A |  |  | N/A |  | 1 | nonsynonymous | 0 |  | 1 | indel | N/A |  | N/A |  | N/A |  |
| 28 | 0 |  | 1 | nonsynonymous |  | N/A |  |  | N/A |  | 1 | nonsynonymous | 0 |  | 0 |  | N/A |  | N/A |  | N/A |  |
| 28 | 0 |  | 1 | nonsynonymous |  | N/A |  |  | N/A |  | 0 |  | 0 |  | 0 |  | N/A |  | N/A |  | N/A |  |
| 28 | 0 |  | 1 | nonsynonymous |  | N/A |  |  | N/A |  | 0 |  | 0 |  | 0 |  | N/A |  | N/A |  | N/A |  |
| 28 | 0 |  | 1 | nonsynonymous |  | N/A |  |  | N/A |  | 0 |  | 0 |  | 0 |  | N/A |  | N/A |  | N/A |  |
| 28 | 0 |  | 1 | nonsynonymous |  | N/A |  |  | N/A |  | 0 |  | 0 |  | 0 |  | N/A |  | N/A |  | N/A |  |
| 28 | 1 | indel | 1 | nonsynonymous |  | N/A |  |  | N/A |  | 0 |  | 0 |  | 0 |  | N/A |  | N/A |  | N/A |  |
| 28 | 0 |  | 1 | nonsynonymous |  | N/A |  |  | N/A |  | 0 |  | 0 |  | 0 |  | N/A |  | N/A |  | N/A |  |
| 28 | 1 | indel | 1 | indel |  | N/A |  |  | N/A |  | 0 |  | 0 |  | 0 |  | N/A |  | N/A |  | N/A |  |
| 28 | 1 | indel | 0 |  |  | N/A |  |  | N/A |  | 0 |  | 0 |  | 0 |  | N/A |  | N/A |  | N/A |  |
| 28 | 0 |  | 1 | nonsynonymous |  | N/A |  |  | N/A |  | 0 |  | 0 |  | 0 |  | N/A |  | N/A |  | N/A |  |
| 28 | 0 |  | 0 |  |  | N/A |  |  | N/A |  | 0 |  | 0 |  | 0 |  | N/A |  | N/A |  | N/A |  |
| 28 | 0 |  | 0 |  |  | N/A |  |  | N/A |  | 0 |  | 0 |  | 0 |  | N/A |  | N/A |  | N/A |  |
| 28 | 0 |  | 1 | nonsynonymous |  | N/A |  |  | N/A |  | 0 |  | 0 |  | 0 |  | N/A |  | N/A |  | N/A |  |
| 28 | 1 | indel | 0 |  |  | N/A |  |  | N/A |  | 0 |  | 0 |  | 0 |  | N/A |  | N/A |  | N/A |  |
| 28 | 0 |  | 0 |  |  | N/A |  |  | N/A |  | 0 |  | 0 |  | 0 |  | N/A |  | N/A |  | N/A |  |
| 28 | 1 | nonsynonymous | 0 |  |  | N/A |  |  | N/A |  | 0 |  | 0 |  | 1 | nonsynonymous | N/A |  | N/A |  | N/A |  |
| 28 | 1 | indel | 1 | nonsynonymous |  | N/A |  |  | N/A |  | 0 |  | 0 |  | 0 |  | N/A |  | N/A |  | N/A |  |
| 28 | 0 |  | 1 | nonsynonymous |  | N/A |  |  | N/A |  | 0 |  | 0 |  | 0 |  | N/A |  | N/A |  | N/A |  |
| 29 | 1 | nonsense | 1 | missense |  | 0 |  |  | 0 |  | N/A |  | N/A |  | N/A |  | N/A | N/A | N/A |  | N/A |  |
| 29 | 1 | indel | 1 | indel |  | 0 |  |  | 0 |  | N/A |  | N/A |  | N/A |  | N/A | N/A | N/A |  | N/A |  |
| 29 | 1 | indel | 1 | missense |  | 0 |  |  | 0 |  | N/A |  | N/A |  | N/A |  | N/A | N/A | N/A |  | N/A |  |
| 29 | 1 | indel | 1 | missense |  | 0 |  |  | 0 |  | N/A |  | N/A |  | N/A |  | N/A | N/A | N/A |  | N/A |  |
| 29 | 1 | nonsense | 1 | missense |  | 0 |  |  | 0 |  | N/A |  | N/A |  | N/A |  | N/A | N/A | N/A |  | N/A |  |
| 29 | 1 | indel | 1 | indel |  | 0 |  |  | 0 |  | N/A |  | N/A |  | N/A |  | N/A | N/A | N/A |  | N/A |  |
| 29 | 1 | nonsense | 1 | missense |  | 0 |  |  | 0 |  | N/A |  | N/A |  | N/A |  | N/A | N/A | N/A |  | N/A |  |
| 29 | 1 | nonsense | 1 | indel |  | 0 |  |  | 0 |  | N/A |  | N/A |  | N/A |  | N/A | N/A | N/A |  | N/A |  |
| 29 | 1 | splice | 1 | missense |  | 0 |  |  | 0 |  | N/A |  | N/A |  | N/A |  | N/A | N/A | N/A |  | N/A |  |
| 29 | 1 | indel | 1 | nonsense |  | 0 |  |  | 0 |  | N/A |  | N/A |  | N/A |  | N/A | N/A | N/A |  | N/A |  |
| 29 | 1 | indel | 1 | missense |  | 0 |  |  | 0 |  | N/A |  | N/A |  | N/A |  | N/A | N/A | N/A |  | N/A |  |
| 29 | 1 | indel | 1 | missense |  | 0 |  |  | 0 |  | N/A |  | N/A |  | N/A |  | N/A | N/A | N/A |  | N/A |  |
| 29 | 1 | splice | 1 | indel |  | 0 |  |  | 0 |  | N/A |  | N/A |  | N/A |  | N/A | N/A | N/A |  | N/A |  |
| 29 | 1 | indel | 1 | missense |  | 0 |  |  | 0 |  | N/A |  | N/A |  | N/A |  | N/A | N/A | N/A |  | N/A |  |
| 29 | 1 | nonsense | 1 | missense |  | 0 |  |  | 0 |  | N/A |  | N/A |  | N/A |  | N/A | N/A | N/A |  | N/A |  |
| 29 | 1 | splice | 1 | missense |  | 0 |  |  | 0 |  | N/A |  | N/A |  | N/A |  | N/A | N/A | N/A |  | N/A |  |
| 29 | 1 | splice | 1 | missense |  | 0 |  |  | 0 |  | N/A |  | N/A |  | N/A |  | N/A | N/A | N/A |  | N/A |  |
| 29 | 1 | nonsense | 1 | missense |  | 0 |  |  | 0 |  | N/A |  | N/A |  | N/A |  | N/A | N/A | N/A |  | N/A |  |
| 29 | 1 | splice | 1 | splice |  | 0 |  |  | 0 |  | N/A |  | N/A |  | N/A |  | N/A | N/A | N/A |  | N/A |  |
| 29 | 1 | nonsense | 1 | indel |  | 0 |  |  | 0 |  | N/A |  | N/A |  | N/A |  | N/A | N/A | N/A |  | N/A |  |
| 29 | 1 | nonsense | 1 | indel |  | 0 |  |  | 0 |  | N/A |  | N/A |  | N/A |  | N/A | N/A | N/A |  | N/A |  |
| 29 | 1 | indel | 1 | splice |  | 0 |  |  | 0 |  | N/A |  | N/A |  | N/A |  | N/A | N/A | N/A |  | N/A |  |
| 29 | 1 | indel | 1 | indel |  | 0 |  |  | 0 |  | N/A |  | N/A |  | N/A |  | N/A | N/A | N/A |  | N/A |  |
| 29 | 1 | indel | 1 | missense |  | 0 |  |  | 0 |  | N/A |  | N/A |  | N/A |  | N/A | N/A | N/A |  | N/A |  |
| 29 | 1 | indel | 1 | indel |  | 0 |  |  | 0 |  | N/A |  | N/A |  | N/A |  | N/A | N/A | N/A |  | N/A |  |
| 29 | 1 | splice | 1 | missense |  | 0 |  |  | 0 |  | N/A |  | N/A |  | N/A |  | N/A | N/A | N/A |  | N/A |  |
| 29 | 1 | splice | 1 | missense |  | 0 |  |  | 0 |  | N/A |  | N/A |  | N/A |  | N/A | N/A | N/A |  | N/A |  |
| 29 | 1 | missense | 1 | missense |  | 0 |  |  | 0 |  | N/A |  | N/A |  | N/A |  | N/A | N/A | N/A |  | N/A |  |
| 29 | 1 | missense | 1 | nonsense |  | 0 |  |  | 1 | missense | N/A |  | N/A |  | N/A |  | N/A | N/A | N/A |  | N/A |  |
| 29 | 1 | indel | 1 | missense |  | 0 |  |  | 1 | missense | N/A |  | N/A |  | N/A |  | N/A | N/A | N/A |  | N/A |  |
| 29 | 1 | indel | 1 | nonsense |  | 0 |  |  | 1 | missense | N/A |  | N/A |  | N/A |  | N/A | N/A | N/A |  | N/A |  |
| 29 | 1 | nonsense | 1 | missense |  | 0 |  |  | 1 | missense | N/A |  | N/A |  | N/A |  | N/A | N/A | N/A |  | N/A |  |
| 29 | 1 | splice | 1 | missense |  | 0 |  |  | 1 | nonsense | N/A |  | N/A |  | N/A |  | N/A | N/A | N/A |  | N/A |  |
| 29 | 1 | nonsense | 1 | missense |  | 0 |  |  | 1 | missense | N/A |  | N/A |  | N/A |  | N/A | N/A | N/A |  | N/A |  |
| 29 | 1 | indel | 0 |  |  | 0 |  |  | 0 |  | N/A |  | N/A |  | N/A |  | N/A | N/A | N/A |  | N/A |  |
| 29 | 1 | indel | 0 |  |  | 0 |  |  | 0 |  | N/A |  | N/A |  | N/A |  | N/A | N/A | N/A |  | N/A |  |
| 29 | 1 | splice | 0 |  |  | 0 |  |  | 0 |  | N/A |  | N/A |  | N/A |  | N/A | N/A | N/A |  | N/A |  |
| 29 | 0 |  | 1 | missense |  | 0 |  |  | 0 |  | N/A |  | N/A |  | N/A |  | N/A | N/A | N/A |  | N/A |  |
| 29 | 0 |  | 1 | missense |  | 0 |  |  | 0 |  | N/A |  | N/A |  | N/A |  | N/A | N/A | N/A |  | N/A |  |
| 29 | 0 |  | 1 | missense |  | 0 |  |  | 0 |  | N/A |  | N/A |  | N/A |  | N/A | N/A | N/A |  | N/A |  |
| 29 | 0 |  | 1 | missense |  | 0 |  |  | 0 |  | N/A |  | N/A |  | N/A |  | N/A | N/A | N/A |  | N/A |  |
| 29 | 0 |  | 1 | missense |  | 0 |  |  | 0 |  | N/A |  | N/A |  | N/A |  | N/A | N/A | N/A |  | N/A |  |
| 29 | 0 |  | 1 | nonsense |  | 0 |  |  | 0 |  | N/A |  | N/A |  | N/A |  | N/A | N/A | N/A |  | N/A |  |
| 29 | 0 |  | 1 | indel |  | 0 |  |  | 0 |  | N/A |  | N/A |  | N/A |  | N/A | N/A | N/A |  | N/A |  |
| 29 | 0 |  | 1 | missense |  | 0 |  |  | 0 |  | N/A |  | N/A |  | N/A |  | N/A | N/A | N/A |  | N/A |  |
| 29 | 0 |  | 1 | nonsense |  | 0 |  |  | 0 |  | N/A |  | N/A |  | N/A |  | N/A | N/A | N/A |  | N/A |  |
| 29 | 0 |  | 1 | nonsense |  | 0 |  |  | 0 |  | N/A |  | N/A |  | N/A |  | N/A | N/A | N/A |  | N/A |  |
| 29 | 0 |  | 1 | indel |  | 0 |  |  | 0 |  | N/A |  | N/A |  | N/A |  | N/A | N/A | N/A |  | N/A |  |
| 29 | 0 |  | 1 | nonsense |  | 0 |  |  | 0 |  | N/A |  | N/A |  | N/A |  | N/A | N/A | N/A |  | N/A |  |
| 29 | 0 |  | 1 | missense |  | 0 |  |  | 0 |  | N/A |  | N/A |  | N/A |  | N/A | N/A | N/A |  | N/A |  |
| 29 | 0 |  | 1 | missense |  | 0 |  |  | 0 |  | N/A |  | N/A |  | N/A |  | N/A | N/A | N/A |  | N/A |  |
| 29 | 0 |  | 1 | missense |  | 0 |  |  | 0 |  | N/A |  | N/A |  | N/A |  | N/A | N/A | N/A |  | N/A |  |
| 29 | 0 |  | 0 |  |  | 0 |  |  | 0 |  | N/A |  | N/A |  | N/A |  | N/A | N/A | N/A |  | N/A |  |
| 29 | 0 |  | 1 | missense |  | 0 |  |  | 1 | missense | N/A |  | N/A |  | N/A |  | N/A | N/A | N/A |  | N/A |  |
| 29 | 0 |  | 1 | missense |  | 0 |  |  | 1 | nonsense | N/A |  | N/A |  | N/A |  | N/A | N/A | N/A |  | N/A |  |
| 29 | 0 |  | 1 | missense |  | 0 |  |  | 1 | missense | N/A |  | N/A |  | N/A |  | N/A | N/A | N/A |  | N/A |  |
| 29 | 0 |  | 0 |  |  | 0 |  |  | 1 | missense | N/A |  | N/A |  | N/A |  | N/A | N/A | N/A |  | N/A |  |
| 29 | 0 |  | 1 | indel |  | 1 | nonsense |  | 1 | missense | N/A |  | N/A |  | N/A |  | N/A | N/A | N/A |  | N/A |  |
| 29 | 0 |  | 1 | missense |  | 1 | nonsense |  | 1 | missense | N/A |  | N/A |  | N/A |  | N/A | N/A | N/A |  | N/A |  |
| 29 | 0 |  | 0 |  |  | 1 | indel |  | 1 | indel | N/A |  | N/A |  | N/A |  | N/A | N/A | N/A |  | N/A |  |
| 29 | 0 |  | 0 |  |  | 1 | missense |  | 1 | missense | N/A |  | N/A |  | N/A |  | N/A | N/A | N/A |  | N/A |  |
| 29 | 0 |  | 1 | splice |  | 1 | nonsense |  | 0 |  | N/A |  | N/A |  | N/A |  | N/A | N/A | N/A |  | N/A |  |
| 29 | 0 |  | 0 |  |  | 1 | missense |  | 0 |  | N/A |  | N/A |  | N/A |  | N/A | N/A | N/A |  | N/A |  |
| 29 | 0 |  | 1 | missense |  | 1 | missense |  | 0 |  | N/A |  | N/A |  | N/A |  | N/A | N/A | N/A |  | N/A |  |
| 29 | 0 |  | 1 | missense |  | 1 | missense |  | 0 |  | N/A |  | N/A |  | N/A |  | N/A | N/A | N/A |  | N/A |  |
| 29 | 0 |  | 1 | missense |  | 0 |  |  | 0 |  | N/A |  | N/A |  | N/A |  | N/A | N/A | N/A |  | N/A |  |
| 29 | 0 |  | 1 | missense |  | 0 |  |  | 0 |  | N/A |  | N/A |  | N/A |  | N/A | N/A | N/A |  | N/A |  |
| 29 | 0 |  | 1 | missense |  | 0 |  |  | 0 |  | N/A |  | N/A |  | N/A |  | N/A | N/A | N/A |  | N/A |  |
| 29 | 0 |  | 1 | missense |  | 0 |  |  | 0 |  | N/A |  | N/A |  | N/A |  | N/A | N/A | N/A |  | N/A |  |
| 29 | 0 |  | 1 | missense |  | 0 |  |  | 0 |  | N/A |  | N/A |  | N/A |  | N/A | N/A | N/A |  | N/A |  |
| 29 | 0 |  | 1 | indel |  | 0 |  |  | 0 |  | N/A |  | N/A |  | N/A |  | N/A | N/A | N/A |  | N/A |  |
| 29 | 0 |  | 1 | nonsense |  | 0 |  |  | 0 |  | N/A |  | N/A |  | N/A |  | N/A | N/A | N/A |  | N/A |  |
| 29 | 0 |  | 1 | missense |  | 0 |  |  | 0 |  | N/A |  | N/A |  | N/A |  | N/A | N/A | N/A |  | N/A |  |
| 29 | 0 |  | 1 | missense |  | 0 |  |  | 0 |  | N/A |  | N/A |  | N/A |  | N/A | N/A | N/A |  | N/A |  |
| 29 | 0 |  | 1 | missense |  | 0 |  |  | 0 |  | N/A |  | N/A |  | N/A |  | N/A | N/A | N/A |  | N/A |  |
| 29 | 0 |  | 0 |  |  | 0 |  |  | 0 |  | N/A |  | N/A |  | N/A |  | N/A | N/A | N/A |  | N/A |  |
| 29 | 0 |  | 0 |  |  | 0 |  |  | 0 |  | N/A |  | N/A |  | N/A |  | N/A | N/A | N/A |  | N/A |  |
| 29 | 0 |  | 0 |  |  | 0 |  |  | 0 |  | N/A |  | N/A |  | N/A |  | N/A | N/A | N/A |  | N/A |  |
| 29 | 0 |  | 0 |  |  | 0 |  |  | 0 |  | N/A |  | N/A |  | N/A |  | N/A | N/A | N/A |  | N/A |  |
| 30 | 1 | stopgain | 1 | nonsynonymou SNV, stopgain |  | 0 |  | 0 | 0 |  | N/A |  | N/A |  | N/A |  | N/A | N/A | N/A |  | N/A |  |
| 30 | 1 | splice | 0 |  |  | 0 |  | 0 | 0 |  | N/A |  | N/A |  | N/A |  | N/A | N/A | N/A |  | N/A |  |
| 30 | 1 | nonsynonymous SNV | 1 | stopgain |  | 0 |  | 0 | 0 |  | N/A |  | N/A |  | N/A |  | N/A | N/A | N/A |  | N/A |  |
| 30 | 1 | stopgain | 1 | frameshift delition |  | 0 |  | 0 | 0 |  | N/A |  | N/A |  | N/A |  | N/A | N/A | N/A |  | N/A |  |
| 30 | 0 |  | 1 | splice |  | 0 |  | 0 | 0 |  | N/A |  | N/A |  | N/A |  | N/A | N/A | N/A |  | N/A |  |
| 30 | 0 |  | 1 | nonsynonymous SNV |  | 0 |  | 0 | 0 |  | N/A |  | N/A |  | N/A |  | N/A | N/A | N/A |  | N/A |  |
| 30 | 0 |  | 0 |  |  | 0 |  | 1 | 1 | nonsynonymous SNV | N/A |  | N/A |  | N/A |  | N/A | N/A | N/A |  | N/A |  |
| 30 | N/A |  | 1 | nonsynonymous SNV |  | N/A | N/A | 1 | 1 | nonsynonymous SNV | N/A |  | N/A |  | N/A |  | N/A | N/A | N/A |  | N/A |  |
| 31 | 0 | N/A | 1 | splice | NoAnnotatedProtChange | 0 | N/A | N/A | N/A |  | N/A |  | N/A |  | N/A |  | N/A | N/A | N/A |  | N/A |  |
| 31 | 1 | N/S | 1 | missense | Pro151Ser | 0 | N/A | N/A | N/A |  | N/A |  | N/A |  | N/A |  | N/A | N/A | N/A |  | N/A |  |
| 31 | 0 | N/A | 0 | N/A | N/A | 0 | N/A | N/A | N/A |  | N/A |  | N/A |  | N/A |  | N/A | N/A | N/A |  | N/A |  |
| 31 | 1 | N/S | 1 | missense | Phe270Cys | 0 | N/A | N/A | N/A |  | N/A |  | N/A |  | N/A |  | N/A | N/A | N/A |  | N/A |  |
| 31 | 1 | N/S | 1 | splice | No　Annotated　Prot Change | 0 | N/A | N/A | N/A |  | N/A |  | N/A |  | N/A |  | N/A | N/A | N/A |  | N/A |  |
| 31 | 1 | N/S | 1 | missense | Ala159Pro | 0 | N/A | N/A | N/A |  | N/A |  | N/A |  | N/A |  | N/A | N/A | N/A |  | N/A |  |
| 31 | 1 | N/S | 1 | missense | Ala138Val | 0 | N/A | N/A | N/A |  | N/A |  | N/A |  | N/A |  | N/A | N/A | N/A |  | N/A |  |
| 31 | 1 | N/S | 1 | missense | Asn131Tyr | 0 | N/A | N/A | N/A |  | N/A |  | N/A |  | N/A |  | N/A | N/A | N/A |  | N/A |  |
| 31 | 0 | N/A | 1 | missense | Gln331His | 0 | N/A | N/A | N/A |  | N/A |  | N/A |  | N/A |  | N/A | N/A | N/A |  | N/A |  |
| 31 | 1 | N/S | 1 | missense | Arg273Cys | 0 | N/A | N/A | N/A |  | N/A |  | N/A |  | N/A |  | N/A | N/A | N/A |  | N/A |  |
| 31 | 1 | N/S | 1 | frameshift deletion | Arg280_Arg282delinsdel | 0 | N/A | N/A | N/A |  | N/A |  | N/A |  | N/A |  | N/A | N/A | N/A |  | N/A |  |
| 31 | 1 | N/S | 1 | nonsense | Gln167Ter | 1 | Frame_Shift_Ins | Glu57GlyfsTer106 | N/A |  | N/A |  | N/A |  | N/A |  | N/A | N/A | N/A |  | N/A |  |
| 31 | 0 | N/A | 1 | missense | Asp281Gly | 0 | N/A | N/A | N/A |  | N/A |  | N/A |  | N/A |  | N/A | N/A | N/A |  | N/A |  |
| 31 | 1 | N/S | 1 | splice | No　AnnotatedProt　Change | 0 | N/A | N/A | N/A |  | N/A |  | N/A |  | N/A |  | N/A | N/A | N/A |  | N/A |  |
| 32 | 1 | frameshift deletion | 1 | missense |  | N/A |  |  | N/A |  | 0 |  | 0 |  | 0 |  | 0 |  | N/A |  | N/A |  |
| 32 | 1 | nonsense | 1 | missense |  | N/A |  |  | N/A |  | 0 |  | 0 |  | 0 |  | 0 |  | N/A |  | N/A |  |
| 32 | 1 | nonsense | 1 | missense |  | N/A |  |  | N/A |  | 0 |  | 0 |  | 0 |  | 0 |  | N/A |  | N/A |  |
| 32 | 1 | splice | 1 | missense |  | N/A |  |  | N/A |  | 0 |  | 0 |  | 0 |  | 0 |  | N/A |  | N/A |  |
| 32 | 1 | splice | 1 | missense |  | N/A |  |  | N/A |  | 0 |  | 0 |  | 0 |  | 0 |  | N/A |  | N/A |  |
| 32 | 1 | splice | 1 | missense |  | N/A |  |  | N/A |  | 1 | inactivating | 1 | inactivating | 0 |  | 0 |  | N/A |  | N/A |  |
| 32 | 1 | nonsense | 1 | missense |  | N/A |  |  | N/A |  | 1 | inactivating | 1 | inactivating | 0 |  | 0 |  | N/A |  | N/A |  |
| 32 | 0 |  | 1 | missense |  | N/A |  |  | N/A |  | 1 | inactivating | 0 |  | 0 |  | 0 |  | N/A |  | N/A |  |
| 32 | 0 |  | 1 | splice |  | N/A |  |  | N/A |  | 1 | inactivating | 0 |  | 1 | inactivating | 0 |  | N/A |  | N/A |  |
| 32 | 0 |  | 1 | missense |  | N/A |  |  | N/A |  | 0 |  | 0 |  | 1 | inactivating | 0 |  | N/A |  | N/A |  |
| 32 | 0 |  | 1 | missense |  | N/A |  |  | N/A |  | 0 |  | 0 |  | 0 |  | 0 |  | N/A |  | N/A |  |
| 32 | 0 |  | 1 | missense |  | N/A |  |  | N/A |  | 0 |  | 0 |  | 0 |  | 0 |  | N/A |  | N/A |  |
| 32 | 0 |  | 1 | missense |  | N/A |  |  | N/A |  | 0 |  | 0 |  | 0 |  | 0 |  | N/A |  | N/A |  |
| 32 | 0 |  | 1 | missense |  | N/A |  |  | N/A |  | 0 |  | 0 |  | 0 |  | 0 |  | N/A |  | N/A |  |
| 32 | 0 |  | 1 | missense |  | N/A |  |  | N/A |  | 0 |  | 0 |  | 0 |  | 0 |  | N/A |  | N/A |  |
| 32 | 0 |  | 1 | missense |  | N/A |  |  | N/A |  | 0 |  | 0 |  | 0 |  | 0 |  | N/A |  | N/A |  |
| 32 | 0 |  | 1 | missense |  | N/A |  |  | N/A |  | 0 |  | 0 |  | 0 |  | 0 |  | N/A |  | N/A |  |
| 32 | 0 |  | 1 | missense |  | N/A |  |  | N/A |  | 0 |  | 0 |  | 0 |  | 0 |  | N/A |  | N/A |  |
| 32 | 0 |  | 0 |  |  | N/A |  |  | N/A |  | 0 |  | 0 |  | 0 |  | 1 | isoform variant | N/A |  | N/A |  |
| 33 | 1 | nonsense | 1 | missense |  | 0 |  |  | N/A |  | 0 |  | N/A |  | N/A |  | N/A | N/A | N/A |  | N/A |  |
| 33 | 0 |  | 0 |  |  | 0 |  |  | N/A |  | 0 |  | N/A |  | N/A |  | N/A | N/A | N/A |  | N/A |  |
| 33 | 0 |  | 1 | missense |  | 0 |  |  | N/A |  | 0 |  | N/A |  | N/A |  | N/A | N/A | N/A |  | N/A |  |
| 33 | 0 |  | 0 |  |  | 0 |  |  | N/A |  | 0 |  | N/A |  | N/A |  | N/A | N/A | N/A |  | N/A |  |
| 33 | 0 |  | 1 | splice |  | 0 |  |  | N/A |  | 0 |  | N/A |  | N/A |  | N/A | N/A | N/A |  | N/A |  |
| 33 | 1 | nonsense | 0 |  |  | 0 |  |  | N/A |  | 0 |  | N/A |  | N/A |  | N/A | N/A | N/A |  | N/A |  |
| 33 | 0 |  | 0 |  |  | 0 |  |  | N/A |  | 0 |  | N/A |  | N/A |  | N/A | N/A | N/A |  | N/A |  |
| 33 | 0 |  | 0 |  |  | 0 |  |  | N/A |  | 0 |  | N/A |  | N/A |  | N/A | N/A | N/A |  | N/A |  |
| 33 | 0 |  | 0 |  |  | 0 |  |  | N/A |  | 0 |  | N/A |  | N/A |  | N/A | N/A | N/A |  | N/A |  |
| 33 | 1 | nonsense | 0 |  |  | 0 |  |  | N/A |  | 0 |  | N/A |  | N/A |  | N/A | N/A | N/A |  | N/A |  |
| 33 | 1 | nonsense | 1 | missense |  | 0 |  |  | N/A |  | 0 |  | N/A |  | N/A |  | N/A | N/A | N/A |  | N/A |  |
| 33 | 0 |  | 0 |  |  | 0 |  |  | N/A |  | 0 |  | N/A |  | N/A |  | N/A | N/A | N/A |  | N/A |  |
| 33 | 1 | nonsense | 1 | missense |  | 0 |  |  | N/A |  | 0 |  | N/A |  | N/A |  | N/A | N/A | N/A |  | N/A |  |
| 33 | 0 |  | 1 | missense |  | 1 | nonsense |  | N/A |  | 0 |  | N/A |  | N/A |  | N/A | N/A | N/A |  | N/A |  |
| 33 | 0 |  | 1 | missense |  | 0 |  |  | N/A |  | 0 |  | N/A |  | N/A |  | N/A | N/A | N/A |  | N/A |  |
| 33 | 0 |  | 1 | nonsense |  | 0 |  |  | N/A |  | 0 |  | N/A |  | N/A |  | N/A | N/A | N/A |  | N/A |  |
| 33 | 0 |  | 1 | missense |  | 0 |  |  | N/A |  | 0 |  | N/A |  | N/A |  | N/A | N/A | N/A |  | N/A |  |
| 33 | 1 | nonsense | 1 | missense |  | 0 |  |  | N/A |  | 0 |  | N/A |  | N/A |  | N/A | N/A | N/A |  | N/A |  |
| 33 | 1 | missense | 1 | nonsense |  | 0 |  |  | N/A |  | 0 |  | N/A |  | N/A |  | N/A | N/A | N/A |  | N/A |  |
| 33 | 0 |  | 1 | missense |  | 1 | nonsense |  | N/A |  | 0 |  | N/A |  | N/A |  | N/A | N/A | N/A |  | N/A |  |
| 33 | 0 |  | 1 | missense |  | 0 |  |  | N/A |  | 0 |  | N/A |  | N/A |  | N/A | N/A | N/A |  | N/A |  |
| 33 | 0 |  | 1 | nonsense |  | 0 |  |  | N/A |  | 0 |  | N/A |  | N/A |  | N/A | N/A | N/A |  | N/A |  |
| 33 | 1 | nonsense | 1 | missense |  | 0 |  |  | N/A |  | 0 |  | N/A |  | N/A |  | N/A | N/A | N/A |  | N/A |  |
| 33 | 1 | missense | 1 | nonsense |  | 0 |  |  | N/A |  | 0 |  | N/A |  | N/A |  | N/A | N/A | N/A |  | N/A |  |
| 33 | 1 | nonsense | 1 | missense |  | 0 |  |  | N/A |  | 0 |  | N/A |  | N/A |  | N/A | N/A | N/A |  | N/A |  |
| 33 | 0 |  | 1 | nonsense |  | 0 |  |  | N/A |  | 0 |  | N/A |  | N/A |  | N/A | N/A | N/A |  | N/A |  |
| 33 | 0 |  | 1 | missense |  | 0 |  |  | N/A |  | 0 |  | N/A |  | N/A |  | N/A | N/A | N/A |  | N/A |  |
| 33 | 0 |  | 1 | missense |  | 0 |  |  | N/A |  | 0 |  | N/A |  | N/A |  | N/A | N/A | N/A |  | N/A |  |
| 33 | 0 |  | 1 | nonsense |  | 0 |  |  | N/A |  | 0 |  | N/A |  | N/A |  | N/A | N/A | N/A |  | N/A |  |
| 33 | 0 |  | 1 | missense |  | 0 |  |  | N/A |  | 0 |  | N/A |  | N/A |  | N/A | N/A | N/A |  | N/A |  |
| 33 | 0 |  | 1 | missense |  | 0 |  |  | N/A |  | 0 |  | N/A |  | N/A |  | N/A | N/A | N/A |  | N/A |  |
| 33 | 0 |  | 1 | missense |  | 0 |  |  | N/A |  | 0 |  | N/A |  | N/A |  | N/A | N/A | N/A |  | N/A |  |
| 33 | 0 |  | 1 | missense |  | 0 |  |  | N/A |  | 0 |  | N/A |  | N/A |  | N/A | N/A | N/A |  | N/A |  |
| 33 | 0 |  | 1 | missense |  | 0 |  |  | N/A |  | 0 |  | N/A |  | N/A |  | N/A | N/A | N/A |  | N/A |  |
| 34 | 1 | truncating | 1 | missense |  | 0 |  |  | 1 | missense | N/A |  | 0 |  | N/A |  | N/A | N/A | N/A |  | N/A |  |
| 34 | 1 | truncating | 1 | missense |  | 0 |  |  | 0 |  | N/A |  | 0 |  | N/A |  | N/A | N/A | N/A |  | N/A |  |
| 34 | 1 | truncating | 1 | truncating |  | 1 | truncating |  | 0 |  | N/A |  | 0 |  | N/A |  | N/A | N/A | N/A |  | N/A |  |
| 34 | 1 | truncating | 1 | missense |  | 1 | missense |  | 0 |  | N/A |  | 0 |  | N/A |  | N/A | N/A | N/A |  | N/A |  |
| 34 | 1 | truncating | 1 | missense |  | 0 |  |  | 0 |  | N/A |  | 0 |  | N/A |  | N/A | N/A | N/A |  | N/A |  |
| 34 | 1 | truncating | 1 | missense |  | 0 |  |  | 0 |  | N/A |  | 0 |  | N/A |  | N/A | N/A | N/A |  | N/A |  |
| 34 | 1 | truncating | 1 | truncating |  | 0 |  |  | 0 |  | N/A |  | 0 |  | N/A |  | N/A | N/A | N/A |  | N/A |  |
| 34 | 1 | truncating | 1 | missense |  | 0 |  |  | 0 |  | N/A |  | 0 |  | N/A |  | N/A | N/A | N/A |  | N/A |  |
| 34 | 1 | truncating | 1 | missense |  | 0 |  |  | 0 |  | N/A |  | 0 |  | N/A |  | N/A | N/A | N/A |  | N/A |  |
| 34 | 1 | truncating | 1 | missense |  | 0 |  |  | 0 |  | N/A |  | 1 | missense | N/A |  | N/A | N/A | N/A |  | N/A |  |
| 34 | 1 | homozygous deletion | 1 | truncating |  | 0 |  |  | 0 |  | N/A |  | 0 |  | N/A |  | N/A | N/A | N/A |  | N/A |  |
| 34 | 0 | N/A | 0 | N/A |  | 0 |  |  | 0 |  | N/A |  | 1 | missense | N/A |  | N/A | N/A | N/A |  | N/A |  |
| 34 | 0 | N/A | 0 | N/A |  | 0 |  |  | 0 |  | N/A |  | 0 |  | N/A |  | N/A | N/A | N/A |  | N/A |  |
| 34 | 0 | N/A | 1 | missense |  | 0 |  |  | 0 |  | N/A |  | 0 |  | N/A |  | N/A | N/A | N/A |  | N/A |  |
| 34 | 1 | truncating | 1 | missense |  | 0 |  |  | 0 |  | N/A |  | 0 |  | N/A |  | N/A | N/A | N/A |  | N/A |  |
| 34 | 1 | truncating | 1 | missense |  | 0 |  |  | 0 |  | N/A |  | 0 |  | N/A |  | N/A | N/A | N/A |  | N/A |  |
| 34 | 1 | truncating | 1 | missense |  | 0 |  |  | 0 |  | N/A |  | 0 |  | N/A |  | N/A | N/A | N/A |  | N/A |  |
| 34 | 1 | truncating | 1 | truncating |  | 1 | truncating |  | 0 |  | N/A |  | 0 |  | N/A |  | N/A | N/A | N/A |  | N/A |  |
| 34 | 1 | truncating | 1 | truncating |  | 0 |  |  | 0 |  | N/A |  | 0 |  | N/A |  | N/A | N/A | N/A |  | N/A |  |
| 34 | 1 | truncating | 1 | missense |  | 0 |  |  | 0 |  | N/A |  | 0 |  | N/A |  | N/A | N/A | N/A |  | N/A |  |
| 34 | 1 | truncating | 1 | missense |  | 0 |  |  | 0 |  | N/A |  | 0 |  | N/A |  | N/A | N/A | N/A |  | N/A |  |
| 34 | 1 | homozygous deletion | 1 | truncating |  | 0 |  |  | 0 |  | N/A |  | 0 |  | N/A |  | N/A | N/A | N/A |  | N/A |  |
| 34 | 1 | truncating | 1 | missense |  | 1 | missense |  | 0 |  | N/A |  | 0 |  | N/A |  | N/A | N/A | N/A |  | N/A |  |
| 34 | 1 | truncating | 1 | missense |  | 0 |  |  | 0 |  | N/A |  | 1 | missense | N/A |  | N/A | N/A | N/A |  | N/A |  |
| 34 | 1 | truncating | 1 | missense |  | 0 |  |  | 1 | missense | N/A |  | 0 |  | N/A |  | N/A | N/A | N/A |  | N/A |  |
| 34 | 1 | missense | 1 | missense |  | 1 | missense |  | 0 |  | N/A |  | 0 |  | N/A |  | N/A | N/A | N/A |  | N/A |  |
| 34 | 1 | truncating | 1 | missense |  | 0 |  |  | 1 | missense | N/A |  | 0 |  | N/A |  | N/A | N/A | N/A |  | N/A |  |
| 34 | 1 | truncating | 1 | missense |  | 0 |  |  | 0 |  | N/A |  | 0 |  | N/A |  | N/A | N/A | N/A |  | N/A |  |
| 34 | 1 | homozygous deletion | 1 | truncating |  | 0 |  |  | 0 |  | N/A |  | 0 |  | N/A |  | N/A | N/A | N/A |  | N/A |  |
| 34 | 1 | homozygous deletion | 1 | missense |  | 0 |  |  | 1 | missense | N/A |  | 0 |  | N/A |  | N/A | N/A | N/A |  | N/A |  |
| 34 | 1 | homozygous deletion | 1 | truncating |  | 0 |  |  | 0 |  | N/A |  | 0 |  | N/A |  | N/A | N/A | N/A |  | N/A |  |
| 34 | 1 | homozygous deletion | 1 | missense |  | 0 |  |  | 0 |  | N/A |  | 1 | missense | N/A |  | N/A | N/A | N/A |  | N/A |  |
| 34 | 1 | truncating | 1 | missense |  | 0 |  |  | 0 |  | N/A |  | 0 |  | N/A |  | N/A | N/A | N/A |  | N/A |  |
| 34 | 1 | truncating | 1 | truncating |  | 0 |  |  | 0 |  | N/A |  | 0 |  | N/A |  | N/A | N/A | N/A |  | N/A |  |
| 34 | 0 | missense | 1 | missense |  | 0 |  |  | 0 |  | N/A |  | 0 |  | N/A |  | N/A | N/A | N/A |  | N/A |  |
| 34 | 0 |  | 1 | missense |  | 0 |  |  | 0 |  | N/A |  | 0 |  | N/A |  | N/A | N/A | N/A |  | N/A |  |
| 34 | 0 |  | 1 | missense |  | 0 |  |  | 1 | truncating | N/A |  | 0 |  | N/A |  | N/A | N/A | N/A |  | N/A |  |
| 34 | 0 |  | 1 | missense |  | 0 |  |  | 1 | truncating | N/A |  | 0 |  | N/A |  | N/A | N/A | N/A |  | N/A |  |
| 34 | 1 | missense | 1 | missense |  | 0 |  |  | 0 |  | N/A |  | 0 |  | N/A |  | N/A | N/A | N/A |  | N/A |  |
| 34 | 1 | truncating | 1 | missense |  | 0 |  |  | 0 |  | N/A |  | 0 |  | N/A |  | N/A | N/A | N/A |  | N/A |  |
| 34 | 0 |  | 1 | truncating |  | 0 |  |  | 0 |  | N/A |  | 0 |  | N/A |  | N/A | N/A | N/A |  | N/A |  |
| 34 | 1 | truncating | 1 | truncating |  | 1 | missense |  | 0 |  | N/A |  | 1 | truncating | N/A |  | N/A | N/A | N/A |  | N/A |  |
| 34 | 0 |  | 1 | missense |  | 0 |  |  | 0 |  | N/A |  | 0 |  | N/A |  | N/A | N/A | N/A |  | N/A |  |
| 34 | 0 |  | 1 | truncating |  | 0 |  |  | 0 |  | N/A |  | 0 |  | N/A |  | N/A | N/A | N/A |  | N/A |  |
| 34 | 0 |  | 0 |  |  | 0 |  |  | 0 |  | N/A |  | 0 |  | N/A |  | N/A | N/A | N/A |  | N/A |  |
| 34 | 0 |  | 0 |  |  | 0 |  |  | 0 |  | N/A |  | 1 | missense | N/A |  | N/A | N/A | N/A |  | N/A |  |
| 35 | 1 | homozygous deletion | 1 | nonsynonymous cording |  | N/A |  |  | N/A |  | N/A |  | 0 |  | N/A |  | N/A |  | N/A |  | N/A |  |
| 35 | 1 | homozygous deletion | 1 | nonsynonymous cording, LOH |  | N/A |  |  | N/A |  | N/A |  | 0 |  | N/A |  | N/A |  | N/A |  | N/A |  |
| 35 | 0 |  | 1 | nonsynonymous cording |  | N/A |  |  | N/A |  | N/A |  | 0 |  | N/A |  | N/A |  | N/A |  | N/A |  |
| 35 | 0 |  | 1 | nonsynonymous cording |  | N/A |  |  | N/A |  | N/A |  | 0 |  | N/A |  | N/A |  | N/A |  | N/A |  |
| 35 | 1 | homozygous deletion | 1 | stop codon, LOH |  | N/A |  |  | N/A |  | N/A |  | 0 |  | N/A |  | N/A |  | N/A |  | N/A |  |
| 35 | 1 | homozygous deletion | 1 | nonsynonymous cording, LOH |  | N/A |  |  | N/A |  | N/A |  | 0 |  | N/A |  | N/A |  | N/A |  | N/A |  |
| 35 | 0 |  | 1 | nonsynonymous cording, LOH |  | N/A |  |  | N/A |  | N/A |  | 0 |  | N/A |  | N/A |  | N/A |  | N/A |  |
| 35 | 0 |  | 1 | nonsynonymous cording, LOH |  | N/A |  |  | N/A |  | N/A |  | 0 |  | N/A |  | N/A |  | N/A |  | N/A |  |
| 35 | 1 | LOH | 1 | stop codon, LOH |  | N/A |  |  | N/A |  | N/A |  | 0 |  | N/A |  | N/A |  | N/A |  | N/A |  |
| 35 | 1 | homozygous deletion | 1 | nonsynonymous cording, LOH |  | N/A |  |  | N/A |  | N/A |  | 0 |  | N/A |  | N/A |  | N/A |  | N/A |  |
| 35 | 0 |  | 1 | nonsynonymous cording |  | N/A |  |  | N/A |  | N/A |  | 0 |  | N/A |  | N/A |  | N/A |  | N/A |  |
| 35 | 0 | homozygous deletion | 1 | nonsynonymous cording |  | N/A |  |  | N/A |  | N/A |  | 0 |  | N/A |  | N/A |  | N/A |  | N/A |  |
| 35 | 1 | frameshift, multiple variants | 1 | nonsynonymous cording, LOH |  | N/A |  |  | N/A |  | N/A |  | 0 |  | N/A |  | N/A |  | N/A |  | N/A |  |
| 35 | 1 | homozygous deletion | 1 | nonsynonymous cording |  | N/A |  |  | N/A |  | N/A |  | 0 |  | N/A |  | N/A |  | N/A |  | N/A |  |
| 35 | 1 | nonsynonymous cording | 1 | stop codon |  | N/A |  |  | N/A |  | N/A |  | 0 |  | N/A |  | N/A |  | N/A |  | N/A |  |
| 35 | 1 | splice site alteration | 1 | nonsynonymous cording, LOH |  | N/A |  |  | N/A |  | N/A |  | 1 | nonsynonymous coding | N/A |  | N/A |  | N/A |  | N/A |  |
| 35 | 0 |  | 1 | nonsynonymous cording |  | N/A |  |  | N/A |  | N/A |  | 1 | nonsynonymous coding | N/A |  | N/A |  | N/A |  | N/A |  |
| 35 | 1 | LOH | 1 | nonsynonymous cording |  | N/A |  |  | N/A |  | N/A |  | 0 |  | N/A |  | N/A |  | N/A |  | N/A |  |
| 35 | 1 | homozygous deletion | 0 |  |  | N/A |  |  | N/A |  | N/A |  | 0 |  | N/A |  | N/A |  | N/A |  | N/A |  |
| 35 | 1 | homozygous deletion | 0 |  |  | N/A |  |  | N/A |  | N/A |  | 1 | nonsynonymous coding | N/A |  | N/A |  | N/A |  | N/A |  |
| 35 | 1 | LOH | 0 |  |  | N/A |  |  | N/A |  | N/A |  | 0 |  | N/A |  | N/A |  | N/A |  | N/A |  |
| 35 | 1 | nonsynonymous cording | 0 |  |  | N/A |  |  | N/A |  | N/A |  | 0 |  | N/A |  | N/A |  | N/A |  | N/A |  |
| 35 | 0 |  | 0 |  |  | N/A |  |  | N/A |  | N/A |  | 0 |  | N/A |  | N/A |  | N/A |  | N/A |  |
| 35 | 0 |  | 1 | LOH |  | N/A |  |  | N/A |  | N/A |  | 1 | splice site alteration | N/A |  | N/A |  | N/A |  | N/A |  |
| 35 | 0 |  | 0 |  |  | N/A |  |  | N/A |  | N/A |  | 0 |  | N/A |  | N/A |  | N/A |  | N/A |  |
| 35 | 1 | LOH | 1 | LOH |  | N/A |  |  | N/A |  | N/A |  | 0 |  | N/A |  | N/A |  | N/A |  | N/A |  |
| 35 | 0 |  | 0 |  |  | N/A |  |  | N/A |  | N/A |  | 0 |  | N/A |  | N/A |  | N/A |  | N/A |  |
| 36 | 0 |  | 1 | N/S |  | N/A |  |  | N/A |  | N/A |  | N/A |  | N/A |  | N/A |  | N/A |  | N/A |  |
| 36 | 0 |  | 0 |  |  | N/A |  |  | N/A |  | N/A |  | N/A |  | N/A |  | N/A |  | N/A |  | N/A |  |
| 36 | 0 |  | 1 | N/S |  | N/A |  |  | N/A |  | N/A |  | N/A |  | N/A |  | N/A |  | N/A |  | N/A |  |
| 36 | 0 |  | 1 | N/S |  | N/A |  |  | N/A |  | N/A |  | N/A |  | N/A |  | N/A |  | N/A |  | N/A |  |
| 36 | 0 |  | 0 |  |  | N/A |  |  | N/A |  | N/A |  | N/A |  | N/A |  | N/A |  | N/A |  | N/A |  |
| 36 | 0 |  | 1 | N/S |  | N/A |  |  | N/A |  | N/A |  | N/A |  | N/A |  | N/A |  | N/A |  | N/A |  |
| 36 | 0 |  | 1 | N/S |  | N/A |  |  | N/A |  | N/A |  | N/A |  | N/A |  | N/A |  | N/A |  | N/A |  |
| 36 | 0 |  | 1 | N/S |  | N/A |  |  | N/A |  | N/A |  | N/A |  | N/A |  | N/A |  | N/A |  | N/A |  |
| 36 | 0 |  | 1 | N/S |  | N/A |  |  | N/A |  | N/A |  | N/A |  | N/A |  | N/A |  | N/A |  | N/A |  |
| 36 | 0 |  | 1 | N/S |  | N/A |  |  | N/A |  | N/A |  | N/A |  | N/A |  | N/A |  | N/A |  | N/A |  |
| 36 | 0 |  | 1 | N/S |  | N/A |  |  | N/A |  | N/A |  | N/A |  | N/A |  | N/A |  | N/A |  | N/A |  |
| 36 | 0 |  | 0 |  |  | N/A |  |  | N/A |  | N/A |  | N/A |  | N/A |  | N/A |  | N/A |  | N/A |  |
| 36 | 0 |  | 0 |  |  | N/A |  |  | N/A |  | N/A |  | N/A |  | N/A |  | N/A |  | N/A |  | N/A |  |
| 36 | 0 |  | 0 |  |  | N/A |  |  | N/A |  | N/A |  | N/A |  | N/A |  | N/A |  | N/A |  | N/A |  |
| 36 | 0 |  | 1 | N/S |  | N/A |  |  | N/A |  | N/A |  | N/A |  | N/A |  | N/A |  | N/A |  | N/A |  |
| 36 | 0 |  | 1 | N/S |  | N/A |  |  | N/A |  | N/A |  | N/A |  | N/A |  | N/A |  | N/A |  | N/A |  |
| 36 | 0 |  | 1 | N/S |  | N/A |  |  | N/A |  | N/A |  | N/A |  | N/A |  | N/A |  | N/A |  | N/A |  |
| 37 | 0 |  | 1 | N/S |  | N/A |  |  | N/A |  | N/A |  | 0 | N/A | 0 | N/A | N/A | N/A | N/A |  | N/A |  |
| 37 | 0 |  | 1 | N/S |  | N/A |  |  | N/A |  | N/A |  | 0 | N/A | 0 | N/A | N/A | N/A | N/A |  | N/A |  |
| 37 | 1 | N/S | 1 | N/S |  | N/A |  |  | N/A |  | N/A |  | 0 | N/A | 0 | N/A | N/A | N/A | N/A |  | N/A |  |
| 37 | 0 |  | 1 | N/S |  | N/A |  |  | N/A |  | N/A |  | 1 | N/S | 0 | N/A | N/A | N/A | N/A |  | N/A |  |
| 37 | 1 | N/S | 0 |  |  | N/A |  |  | N/A |  | N/A |  | 0 | N/A | 0 | N/A | N/A | N/A | N/A |  | N/A |  |
| 37 | 1 | N/S | 1 | N/S |  | N/A |  |  | N/A |  | N/A |  | 0 | N/A | 0 | N/A | N/A | N/A | N/A |  | N/A |  |
| 37 | 0 |  | 1 | N/S |  | N/A |  |  | N/A |  | N/A |  | 0 | N/A | 0 | N/A | N/A | N/A | N/A |  | N/A |  |
| 37 | 1 | N/S | 1 | N/S |  | N/A |  |  | N/A |  | N/A |  | 0 | N/A | 0 | N/A | N/A | N/A | N/A |  | N/A |  |
| 37 | 1 | N/S | 1 | N/S |  | N/A |  |  | N/A |  | N/A |  | 0 | N/A | 1 | N/S | N/A | N/A | N/A |  | N/A |  |
| 37 | 0 |  | 0 |  |  | N/A |  |  | N/A |  | N/A |  | 0 | N/A | 0 | N/A | N/A | N/A | N/A |  | N/A |  |
| 37 | 0 |  | 1 | N/S |  | N/A |  |  | N/A |  | N/A |  | 0 | N/A | 0 | N/A | N/A | N/A | N/A |  | N/A |  |
| 37 | 0 |  | 1 | N/S |  | N/A |  |  | N/A |  | N/A |  | 0 | N/A | 1 | N/S | N/A | N/A | N/A |  | N/A |  |
| 37 | 0 |  | 1 | N/S |  | N/A |  |  | N/A |  | N/A |  | 0 | N/A | 0 | N/A | N/A | N/A | N/A |  | N/A |  |
| 37 | 0 |  | 1 | N/S |  | N/A |  |  | N/A |  | N/A |  | 0 | N/A | 0 | N/A | N/A | N/A | N/A |  | N/A |  |
| 38 | 1 | CNV | 1 | CNV |  | 0 |  |  | 1 | missense | 0 |  | N/A |  | N/A |  | N/A | N/A | N/A |  | N/A |  |
| 38 | 1 | nonsense | 1 | nonsense |  | 1 | missense |  | 1 | missense | 1 | missense | N/A |  | N/A |  | N/A | N/A | N/A |  | N/A |  |
| 38 | 0 |  | 1 | frame shift InsDel |  | 0 |  |  | 0 |  | 0 |  | N/A |  | N/A |  | N/A | N/A | N/A |  | N/A |  |
| 38 | 1 | CNV | 1 | CNV |  | 0 |  |  | 0 |  | 0 |  | N/A |  | N/A |  | N/A | N/A | N/A |  | N/A |  |
| 38 | 1 | nonsense | 1 | missense |  | 0 |  |  | 0 |  | 1 | missense | N/A |  | N/A |  | N/A | N/A | N/A |  | N/A |  |
| 38 | 0 |  | 1 | CNV |  | 1 | CNV |  | 0 |  | 0 |  | N/A |  | N/A |  | N/A | N/A | N/A |  | N/A |  |
| 38 | 1 | nonsense | 1 | frame shift InsDel |  | 0 |  |  | 1 | nonsense | 0 |  | N/A |  | N/A |  | N/A | N/A | N/A |  | N/A |  |
| 38 | 1 | nonsense | 1 | frame shift InsDel |  | 0 |  |  | 0 |  | 0 |  | N/A |  | N/A |  | N/A | N/A | N/A |  | N/A |  |
| 38 | 1 | CNV | 1 | CNV |  | 0 |  |  | 0 |  | 0 |  | N/A |  | N/A |  | N/A | N/A | N/A |  | N/A |  |
| 38 | 1 | splice | 1 | splice |  | 0 |  |  | 0 |  | 0 |  | N/A |  | N/A |  | N/A | N/A | N/A |  | N/A |  |
| 38 | 0 |  | 1 | missense |  | 0 |  |  | 0 |  | 0 |  | N/A |  | N/A |  | N/A | N/A | N/A |  | N/A |  |
| 38 | 0 |  | 1 | missense |  | 0 |  |  | 0 |  | 0 |  | N/A |  | N/A |  | N/A | N/A | N/A |  | N/A |  |
| 38 | 0 |  | 1 | missense |  | 0 |  |  | 0 |  | 0 |  | N/A |  | N/A |  | N/A | N/A | N/A |  | N/A |  |
| 38 | 0 |  | 1 | missense |  | 0 |  |  | 1 | missense | 0 |  | N/A |  | N/A |  | N/A | N/A | N/A |  | N/A |  |
| 38 | 0 |  | 1 | CNV |  | 0 |  |  | 0 |  | 0 |  | N/A |  | N/A |  | N/A | N/A | N/A |  | N/A |  |
| 38 | 0 |  | 1 | missense |  | 0 |  |  | 0 |  | 1 | nonsense | N/A |  | N/A |  | N/A | N/A | N/A |  | N/A |  |
| 38 | 0 |  | 1 | nonsense |  | 0 |  |  | 0 |  | 0 |  | N/A |  | N/A |  | N/A | N/A | N/A |  | N/A |  |
| 38 | 0 |  | 1 | splice |  | 0 |  |  | 0 |  | 0 |  | N/A |  | N/A |  | N/A | N/A | N/A |  | N/A |  |
| 38 | 0 |  | 1 | missense |  | 0 |  |  | 0 |  | 0 |  | N/A |  | N/A |  | N/A | N/A | N/A |  | N/A |  |
| 38 | 0 |  | 1 | missense |  | 0 |  |  | 0 |  | 0 |  | N/A |  | N/A |  | N/A | N/A | N/A |  | N/A |  |
| 38 | 0 |  | 1 | missense |  | 0 |  |  | 0 |  | 0 |  | N/A |  | N/A |  | N/A | N/A | N/A |  | N/A |  |
| 38 | 1 | missense | 0 |  |  | 0 |  |  | 1 | missense | 1 | missense | N/A |  | N/A |  | N/A | N/A | N/A |  | N/A |  |
| 38 | 0 |  | 0 |  |  | 0 |  |  | 0 |  | 1 | nonsense | N/A |  | N/A |  | N/A | N/A | N/A |  | N/A |  |
| 38 | 0 |  | 0 |  |  | 0 |  |  | 0 |  | 0 |  | N/A |  | N/A |  | N/A | N/A | N/A |  | N/A |  |
| 38 | 0 |  | 0 |  |  | 0 |  |  | 0 |  | 0 |  | N/A |  | N/A |  | N/A | N/A | N/A |  | N/A |  |
| 38 | 0 |  | 0 |  |  | 0 |  |  | 0 |  | 0 |  | N/A |  | N/A |  | N/A | N/A | N/A |  | N/A |  |
| 38 | 0 |  | 0 |  |  | 0 |  |  | 0 |  | 0 |  | N/A |  | N/A |  | N/A | N/A | N/A |  | N/A |  |
| 38 | 0 |  | 0 |  |  | 0 |  |  | 0 |  | 0 |  | N/A |  | N/A |  | N/A | N/A | N/A |  | N/A |  |

N/A, not available; N/S, not specified; 0, -ve; 1,+ve

References 13, 65 and 66 are only the number of cases and details are unknown.
